# Development of a novel *Artemia* eggshell-zirconium nanocomposite for efficient fluoride removal

**DOI:** 10.1371/journal.pone.0244711

**Published:** 2021-01-04

**Authors:** Wen Zhang, Yuqin Mao, Yin Lu

**Affiliations:** College of Biology and Environmental Engineering, Zhejiang Shuren University, Hangzhou, China; Qatar University, QATAR

## Abstract

Fluoride pollution in water has attracted widespread concern worldwide. In this study, an *Artemia* eggshell-zirconium (Aes-Z) nanocomposite has been used for fluoride removal. Material characterization results showed that nano-ZrO_2_ was immobilized on the inner surface of the *Artemia* eggshell, and there was no pore blockage on the composite material. Various parameters influencing on the fluoride removal, including treatment time, composite dosage, pH, initial fluoride concentration, and other anions, were analyzed. The removal efficiency of the composite material was better than that of the single zirconia material. The removal percentage of fluoride reached 93% in 30 min with an initial fluoride concentration of 10 mg/L and a nanocomposite dosage of 8.0 g/L. The composite material had a high removal efficiency for fluoride in the pH region 4.0–10.0. The adsorption of fluoride was not influenced by the common anions (e.g., Cl^-^, SO_4_^2-^, and NO_3_^-^) in water. The regeneration revealed that the Aes-Z composite material could be reused and remove fluoride effectively in four cycles. The pseudo-second-order rate model adequately represented the adsorption kinetics of the Aes-Z composite material. A possible, defluoridation mechanism of the Aes-Z composite material was also proposed. This study demonstrates that Aes-Z is a promising adsorbent material for fluoride removal.

## Introduction

Fluoride (F^-^) pollution in drinking water has attracted wide attention worldwide because its excessive content causes health problems, including fluorosis, osteoporosis, cancer, arthritis, brain injury, and neurological diseases [1, 2]. According to the World Health Organization (WHO) standard [3], the fluoride concentration of drinking water should be lower than 1.5 mg/L. In China, this value must be controlled below 1.0 mg/L [4]. Although groundwater is an important part of drinking water resources, its fluoride concentration ranges from 1 ppm to more than 35 ppm [5]. Approximately, 300 million people worldwide are affected by fluoride pollution from drinking water sources [6, 7]. In China, over 60 million people suffer from health hazards caused by excessive fluoride content in drinking water [8]. Therefore, developing low-cost and highly efficient fluoride removal technology is crucial.

Several defluorination technologies, such as adsorption [9, 10], ion exchange [11, 12], precipitation [13], membrane separation [14, 15], electrocoagulation flotation [16], and electrodialysis [17], have been developed and evaluated. Among these methods, adsorption is still the first option owing to its high removal rate, easy operation and cost-effectiveness [18–20]. However, developing a suitable adsorbent with acceptable adsorption capacity, high regeneration efficiency, and strong adaptability remains difficult [21]. New adsorption materials are considered as the main direction of research on the defluorination of drinking water for practical applications [22].

Zirconium has attracted increasing attention because of its high binding affinity with fluoride and acceptable costs [10]. Zirconium-based materials have shown promising prospects in the field of fluoride removal, but these materials usually exist in the form of powders or precipitated suspensions. For example, zirconia (ZrO_2_) can be used as an efficient adsorption material for defluorination treatment, where its insoluble complex is formed to achieve fluoride removal. However, drawbacks such as poor separation and low hydraulic conductivity limit its further application [23]. Coating, loading, impregnating, or encapsulating Zr-based adsorbents in some carriers have become important ways to overcome these defects. A hybrid material prepared by combining shellac and zirconium can be selected as an adsorbent to effectively defluorinate drinking water in the pH range of 6–7 [24]. Meanwhile, results have shown that the adsorption efficiency of this shellac zirconium hybrid material for fluoride was 70–97% after the first to sixth cycles of intermittent operation with a small effect of co-ions on the adsorption capacity [24]. Zr-based cellulose is also suitable for fluoride removal from aqueous solutions in weakly acidic pH 4.5–5.5, where the adsorption capacity of the new Zr-impregnated cellulose adsorbent has reached 4.95 mg/g [25]. Therefore, exploiting the combination of carriers (especially materials obtained from natural sources) and zirconium is a suitable way to develop composite materials, which are both efficient and economical for removing fluoride from water. The use of materials from natural sources is crucial owing to environmental safety and the potential to reduce the cost of material preparation.

*Artemia*, belonging to Crustacea, is widely distributed in salt fields and high salt lakes worldwide. China has nearly a thousand of large- and medium-sized inland salt lakes, with many *Artemia* resources. *Artemia* eggs can survive in freezing, anoxia, and other extreme environments, as well as resist drying, high heat, radiation, and high salt for extended periods, owing to the protective effect of their shells. In aquaculture, *Artemia* eggshells are discarded as waste. However, *Artemia* eggshells as a biomaterial have many advantages, such as good biocompatibility, environmental friendliness, and excellent stability. Moreover, the effective use of waste resources is conducive to sustainable societies. The special pore structure of *Artemia* eggshell has also been reported [26]. Research has shown that the *Artemia* eggshells have a unique pore structure, showing a gradually shrinking pore. Its inner pore is a nano-pore, which can promote the distribution of nanoparticles and has the effect of a nano-template. The outer pore is macro-pore, which significantly improves the efficiency of adsorption and mass transfer. Compared with traditional materials, *Artemia* eggshells not only overcomes the problem of a low mass transfer rate in micropores, but also the problems of particle agglomeration, pore blockage, and small adsorption capacity. They have been previously used as carrier materials in environmental treatments. For example, the *Artemia* eggshell-supported TiO_2_ nanocomposite can be used for the efficient removal of formaldehyde [27]. This also indicates that the combination of *Artemia* eggshell and nanoparticles is feasible. On the adsorption of fluoride ions, nano-template-supported zirconium material can also be formed through the special pore structure inside the *Artemia* eggshell. The specific surface area of zirconium can be increased and its adsorption capacity improved. Further, the external macrospore of *Artemia* eggshells can promote the transfer efficiency of fluoride ions and avoid the influence of structural resistance on the adsorption process. Therefore, the application of *Artemia* eggshell as a carrier material in the field of fluoride removal is promising.

In this paper, we report the preparation of a new Aes-Z material used for fluoride removal. The morphology and structure of the prepared materials were characterized using various analytical methods, such as X-ray diffraction (XRD), scanning electron microscopy (SEM), transmission electron microscopy (TEM), and Fourier-transform infrared (FTIR) spectroscopy. Various parameters for fluoride removal, including the composite dosage, treatment time, pH, initial fluoride concentration, and other anions (Cl^-^, SO_4_^2-^, and NO_3_^-^), were analyzed. The reusability of the Aes-Z material was studied by regeneration experiments and the adsorption kinetics of the Aes-Z material are discussed. The mechanisms of fluoride removal by the Aes-Z material were also analyzed.

## Materials and methods

### Materials

*Artemia* eggs were obtained from Zhejiang, PR China. All the analytical grade reagents, namely, zirconium oxychloride, anhydrous ethanol, sodium fluoride, sodium chloride, sodium hydroxide, sodium carbonate, sodium bicarbonate, sulfuric acid, ethylene diamine tetraacetic acid disodium, and hydrochloric acid, were purchased from the Tianjin Chemical Reagent Factory. To avoid ion interference, deionized water was employed in this study. The standard fluoride solution (NaF, 1 g/L) was prepared first and then diluted before use.

### Preparation of Artemia eggshell (Aes)

The eggshells were collected by a sieve with a pore diameter of 0.25–0.3 mm when the *Artemia* eggs had been hatched for approximately 24 h. Then, the collected eggshells were poured into a conical flask (with deionized water) and shaken for 30–45 min to remove impurities. Then the shells were kept for 15 min and collected again by the sieve. The collected shells were thoroughly washed to a neutral pH with deionized water and then dried.

### Preparation of Artemia eggshell -zirconium (Aes-Z) material using in situ precipitation method

Zirconium oxychloride (ZrOCl_2_, 15 g) was added to 150 mL of deionized water. The solution was stirred until the solid completely dissolved. Then, 20% (w/w) sodium hydroxide was added to adjust the pH to 7.0. After filtration and washing, the solid was dried to obtain zirconia. A total of 6 g prepared zirconia were mixed with 5 g of eggshells. Then, the mixture was added into 150 mL anhydrous ethanol solution for ultrasonic treatment at 40 kHz for 4 h and magnetic stirring (200 rpm) for 5 h. After the in situ precipitation reaction, the eggshells were completely sunk to the bottom of the container. After filtration, washing with distilled water to neutrality, and oven drying (333 K, 2 h, air), the Aes-Z material was prepared and sealed for use. The zirconia content of the prepared Aes-Z materials was then measured.

### Adsorption experiments

Batch adsorption experiments were carried out in this study. All adsorption experiments were performed at 298 K. The contents were shaken at 200 rpm. The blank test was set in the same operation mode without adding adsorbent or other materials. The results showed that the concentration of fluoride did not change in the blank test. After adsorption, the suspension was centrifuged, and the fluoride concentration of the supernatant was measured to calculate the removal efficiency of fluoride ions. Different amounts of Aes-Z material (1.0, 2.0, 4.0, 6.0, 8.0, and 10.0 g/L) were added to the solutions (10 mg/L, pH = 6) to investigate the influence of dosage on the fluoride removal efficiency. The adsorption treatment time was 30 min. According to this experiment, the optimal dosage of the Aes-Z adsorbent was determined. Fluoride removal experiments were also performed at different treatment times (0 min, 15 min, 30 min, 1 h, 3 h, 6 h, 9 h, 12 h, and 24 h) to determine the most appropriate adsorption time. As there are few strong alkaline and acid water bodies in the actual environment, the initial pH of the fluoride solution was adjusted to 4–10 by a hydrochloric acid or sodium hydroxide solution during the fluoride removal experiments. The influence of fluoride concentration on the removal efficiency was studied by varying the fluoride ion concentration (2, 5, 10, 20, 30, 40 mg/L). Other anions with different concentrations were added to the solutions with a fluoride concentration of 10 mg/L to analyze the effect of other co-existing anions on the adsorption process. The concentration of other anions was set according to the water quality of the natural water. The adsorption kinetics research of the Aes-Z material was conducted as follows: 8.0 g/L Aes-Z was poured into the fluoride solution (10 mg/L, pH = 6), and the concentration of fluoride was measured at different reaction times. All the above experiments were repeated three times and the average data were calculated and reported.

### Desorption and reuse experiment

Four adsorption and desorption cycles were used to test the material reuse. In each cycle, the composite material was added to the fluoride solution. The initial fluoride concentration of the solution was also set to 10 mg/L. After shaking for 30 min, the material was washed in 200 mL of a mixture solution of 3% NaOH and 2% NaCl for 4 h under the shaking at 160 rpm. After desorption, the material to obtain the regenerated adsorption material. Then, the adsorption material was reused in the next cycle. The reusability of the material was evaluated by calculating the adsorption capacity of the material for fluoride under different cycles of use.

#### Analysis methods

The concentration of fluoride was detected by ion chromatography (ics-5000, Dionex, USA). A mixed solution of 4.5-mmol sodium carbonate and 1.4-mmol sodium bicarbonate was selected as the mobile phase, and a standard curve was constructed. The content of zirconia in the composite material was measured as follows: 1.00 g of Aes-Z adsorbent were placed in a beaker and mixed with 3.00 g of ammonium sulfate and 7.00 mL of concentrated sulfuric acid. Then, the mixture was heated until the solid was completely dissolved. The mixture was then cooled to a fixed volume with diluted hydrochloric acid. The next step was normal titration. A solution of 0.02-M EDTA-2Na, was solution as the titrant and 0.2% xylenol orange was used as an indicator. The content of zirconia was calculated using the volume of the titrant. Further, the content of zirconia in the composite was also measured by inductively coupled plasma atomic emission spectrometry (icap6300, radial, USA).

SEM-energy dispersive X-ray spectroscopy (EDS) (s-4800 II, Hitachi, Japan/Horiba, Japan) and TEM (jem-2010fx, Jeol, USA) were used to analyze the morphological and structural characteristics of the materials. XRD (dx-6000, Shimadzu, Japan) was used to analyze the materials. The zeta potential of the materials was measured using a zeta potential analyzer (Nano ZS90, Malvern Company, UK). The FTIR analyses for the adsorbent were carried out using an FTIR spectrometer (Nicolet iS10, Thermo Fisher Scientific). The specific surface area of the material was measured using a specific surface area tester (F-Sorbx 400; Beijing Jinaipu Technology Company, China). All the measurement experiments were conducted at room temperature.

Simultaneously, the removal percentage of fluoride and adsorption capacity (q_e_, mg/g) of the material were calculated as follows [28]:

$$\text{Removal}\;\text{percentage}\left(\text{\%}\right)=\frac{C_{0}-C_{f}}{C_{0}}\times 100\%$$ (1)

$$q_{e}=\frac{\left(C_{0}-C_{f}\right)V}{W}$$ (2)

where *C*_*0*_ (mg/L) is the initial concentration of fluoride, *C*_*f*_ (mg/L) is the concentration of fluoride at the contact time t, *V* is the volume (L) of fluoride solution, and *W* is the dry weight (g) of the adsorption material used.

## Results

### Characterization results of the Aes-Z material

ZrO_2_ is the main functional adsorber in composite materials, so the content of zirconia loaded on the composite materials should be investigated before material application. Based on the analysis methods, the content of zirconia in the prepared composite was 42.20%.

**Fig 1:**
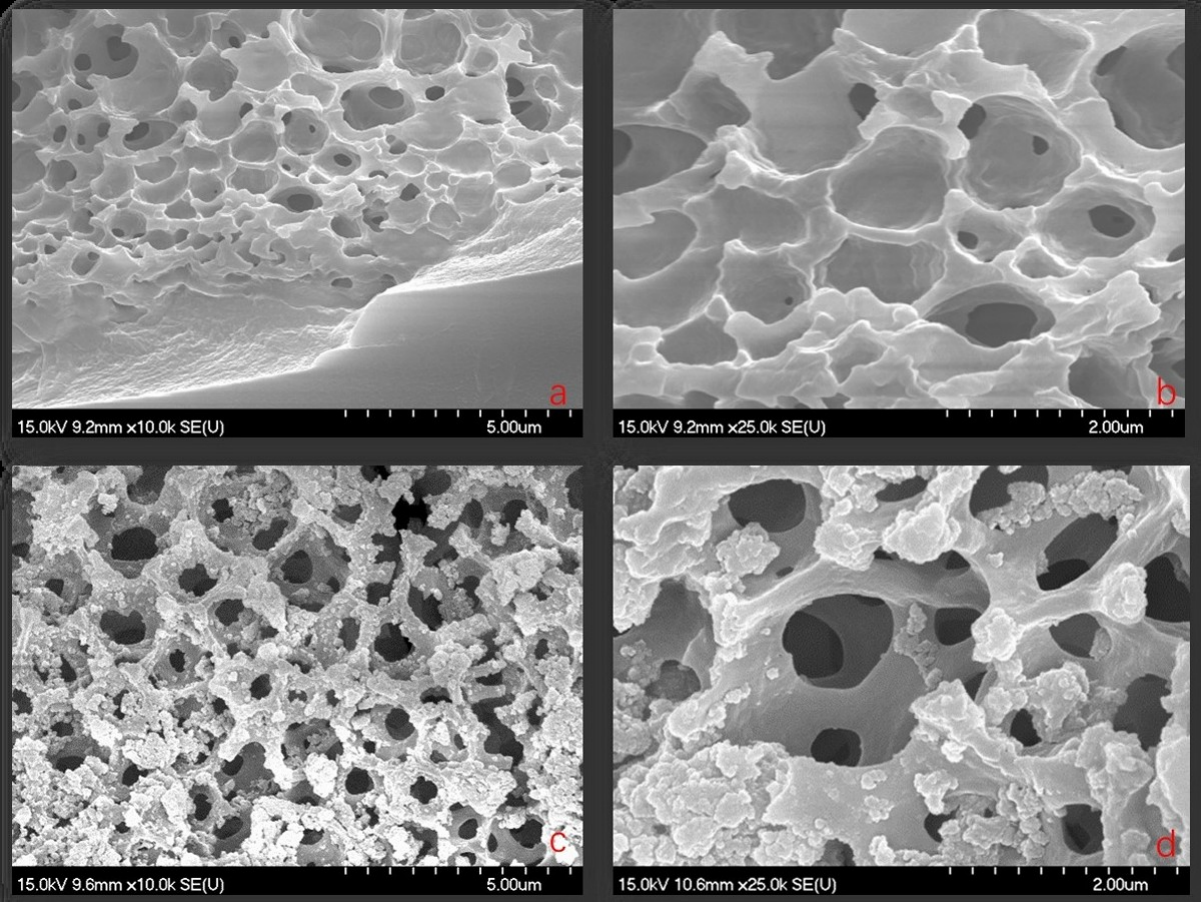
SEM patterns of *Artemia* eggshell and *Artemia* eggshell-zirconium (Aes-Z) material. (a,b: *Artemia* eggshell; c,d: *Artemia* eggshell-zirconium (Aes-Z) material).

**Fig 2:**
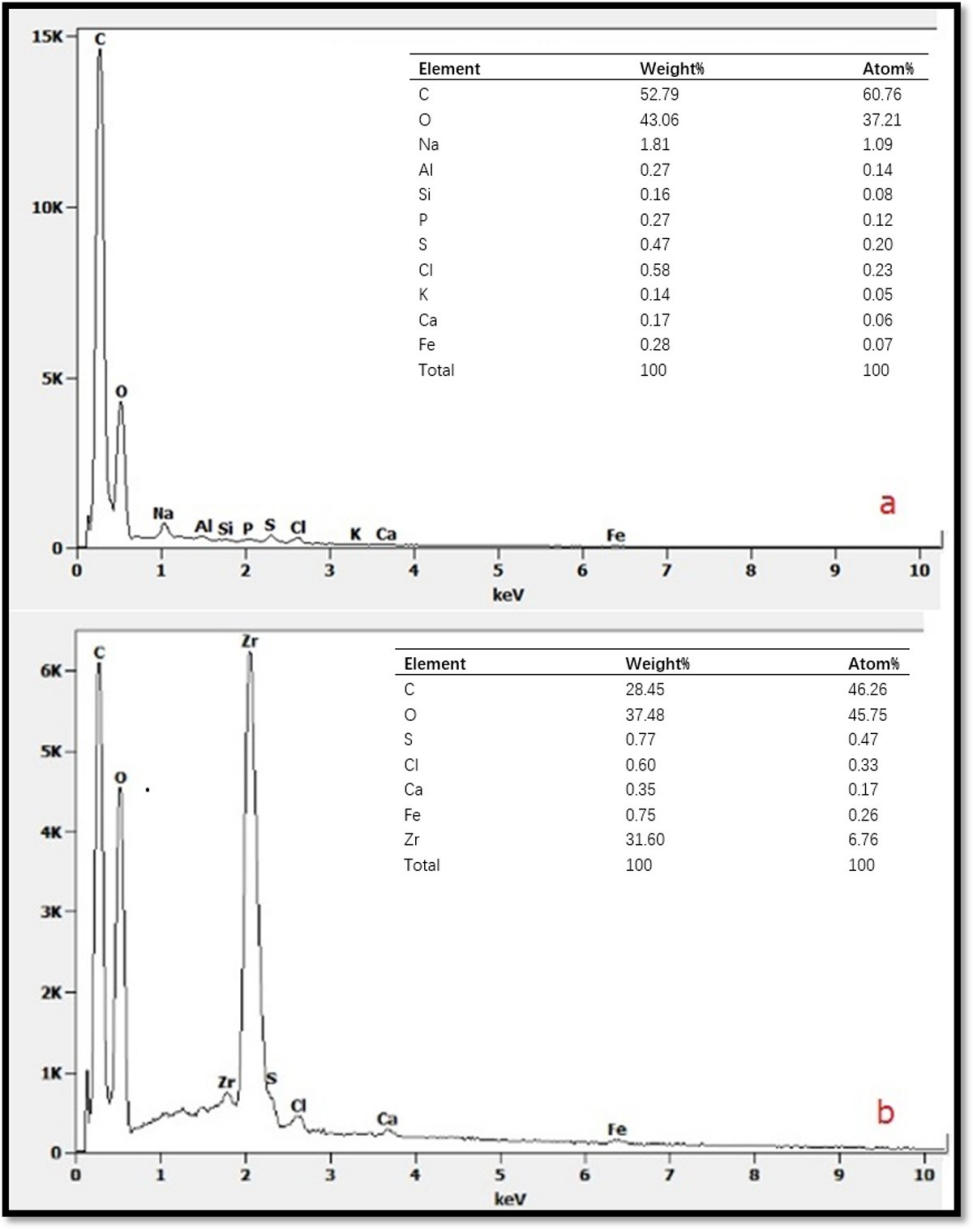
EDS patterns of *Artemia* eggshell and *Artemia* eggshell-zirconium (Aes-Z) material. (a: *Artemia* eggshell;b: *Artemia* eggshell-zirconium (Aes-Z) material).

**Fig 3:**
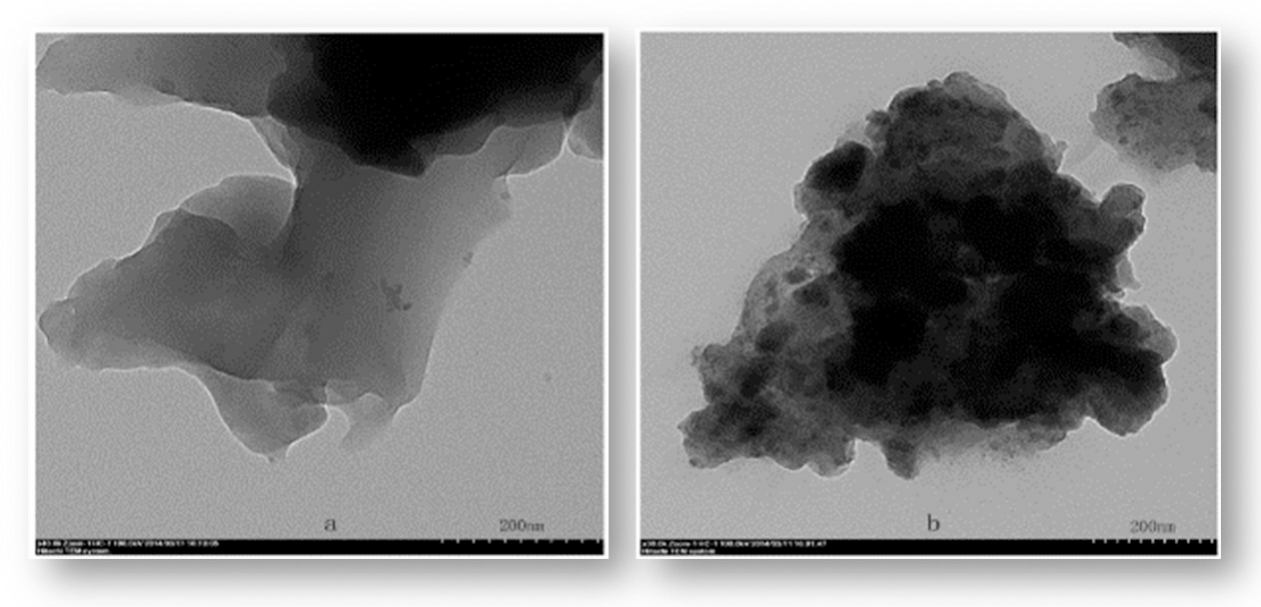
TEM patterns of *Artemia* eggshell and *Artemia* eggshell-zirconium (Aes-Z) material. (a: *Artemia* eggshell; b: *Artemia* eggshell-zirconium (Aes-Z) material).

Figs 1 and 2 show SEM and EDS images of *Artemia* eggshells and the Aes-Z composite, respectively. There were smooth pores in the inner part of the *Artemia* eggshell (Fig 1A and 1B), and the outer pores were larger than the inner ones. Many particles appeared on the surface of the Aes-Z) material (Fig 1C and 1D). According to the EDS analysis of the *Artemia* shells, the elements of *the Artemia* shell contain C, O, Na, Al, P, S, Mg, K, Ca, etc, in which C and O are the main components. There was no Zr in the *Artemia* eggshell samples (Fig 2A), but the Zr element content was high in the Aes-Z material (Fig 2B). The mass ratio of Zr element in the sample scattering area was second (31.6%). The TEM results show that, before zirconia was fixed (Fig 3A), there was no black speckled particle in the eggshell, and the transparency was high. After zirconia was fixed (Fig 3B), there were noticeable black speckled particles in the eggshell, and the maximum size of zirconia particles fixed to the inner surface of the eggshell pore was approximately 10–50 nm. The specific surface area of the composite was 41.2 m^2^g^-1^, and there was a large amount of adsorption space.

**Fig 4:**
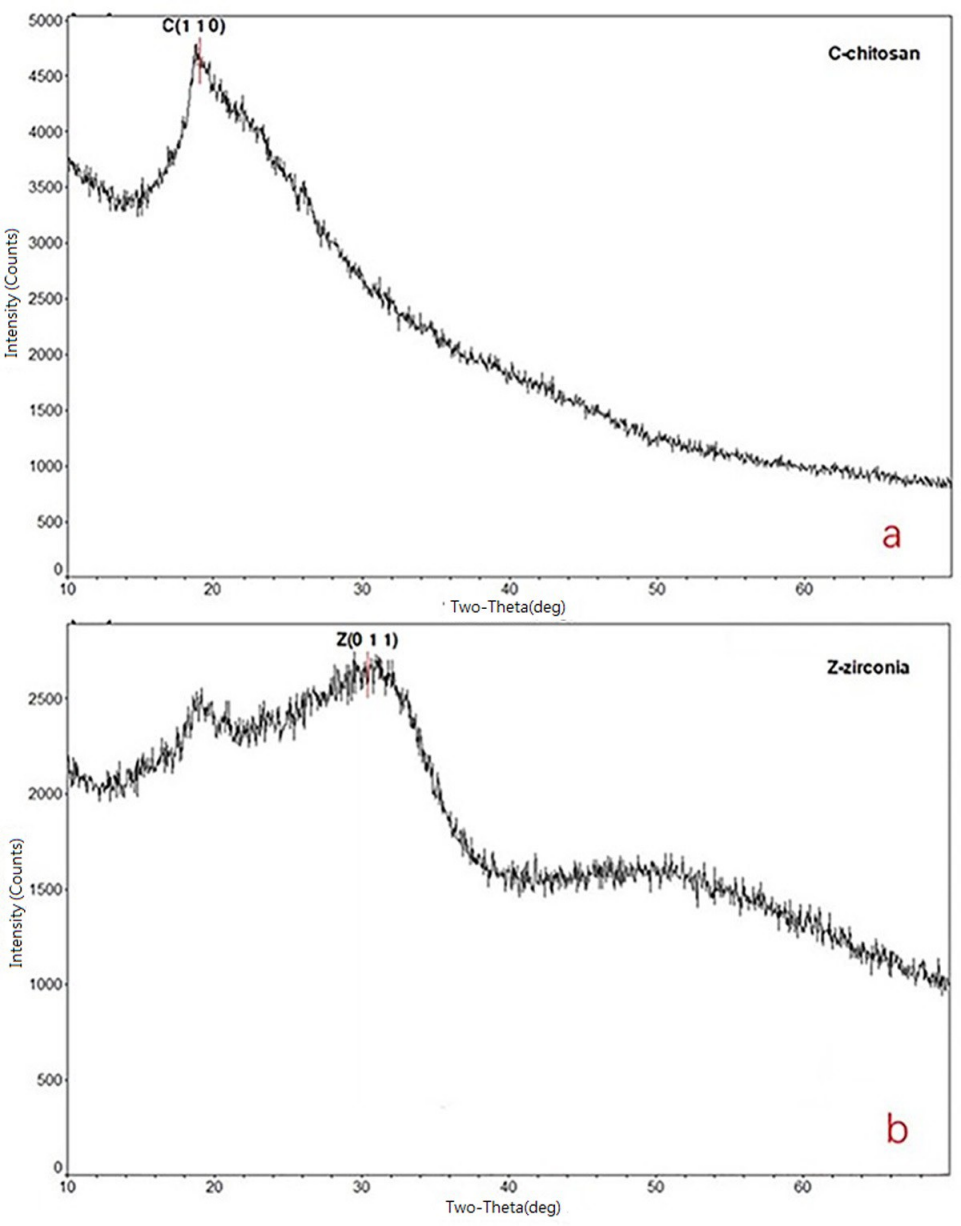
XRD patterns of *Artemia* eggshell and *Artemia* eggshell-zirconium (Aes-Z) material. (a: *Artemia* eggshell; b: *Artemia* eggshell-zirconium (Aes-Z) material).

The XRD analysis of the Aes-Z composition is shown in Fig 4. There were many peaks in the eggshell as because it belongs to the organism. The X-ray diffraction pattern of the *Artemia* shell showed that the broad peak of 2θ–20° was consistent with that of chitosan. The broad reflection at 2θ–20° was indexed to the (1 1 0) reflections of chitosan (form II crystal form) [29]. In the Aes-Z composite, it was found that the peak position matches the standard peak position of zirconia, and that the peak strength was essentially the same. The broad reflection at 2θ–30.8° of the Aes-Z composite was indexed to the (0 1 1) reflections of zirconia (JCPDS, file no. 83–0810) [30].

### Adsorption of F- by the *Artemia* eggshell-zirconium (Aes-Z) material

#### Effect of adsorption treatment time

**Fig 5:**
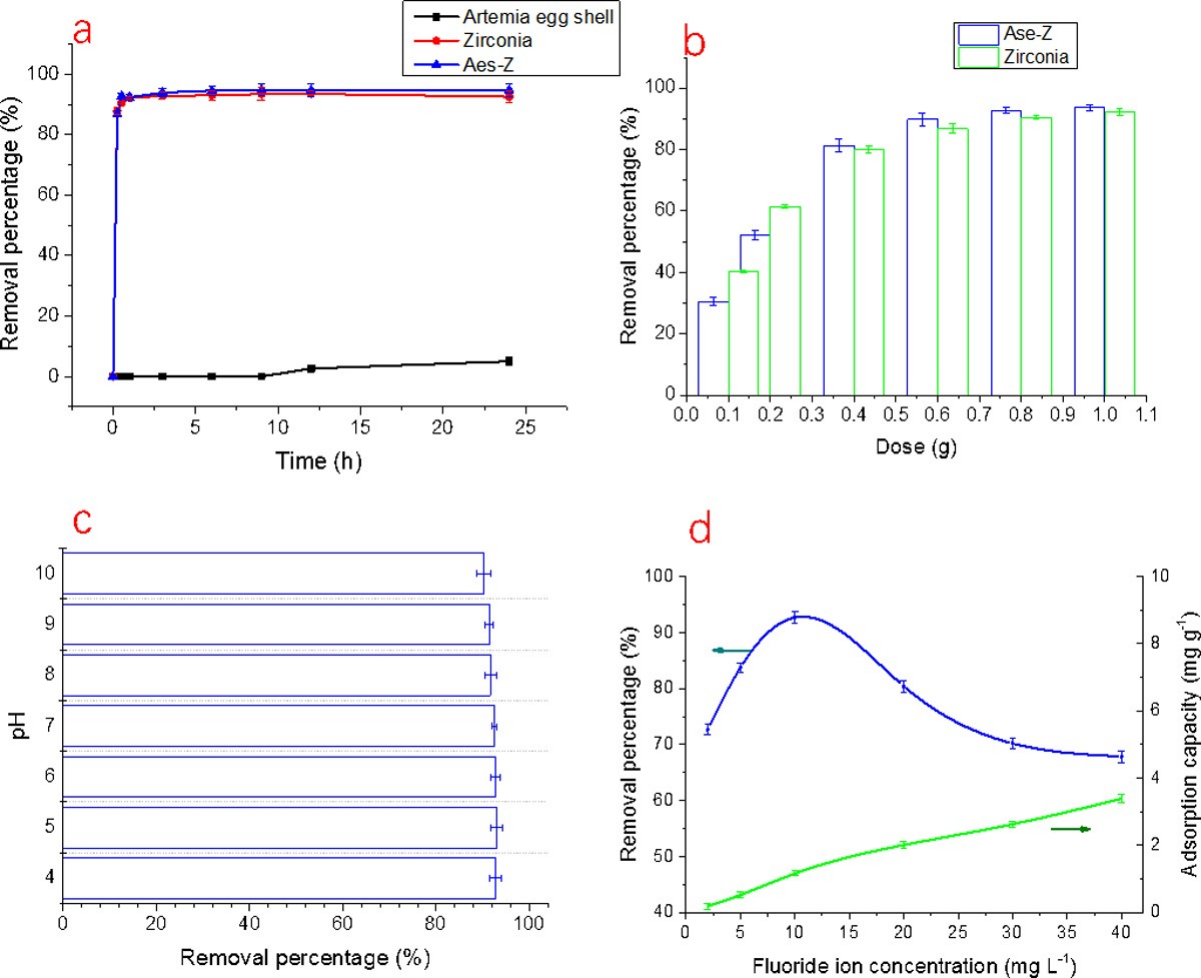
Analysis of factors affecting the adsorption of fluoride. (a: Effect of treatment time on fluoride removal; b: Effect of dosage on fluoride removal; c: Effect of pH on fluoride removal; d: Effect of fluoride ion concentration on fluoride removal).

First, three types of materials (i.e. *Artemia* eggshell, the zirconia, and Aes-Z composite) were added to the fluoride-containing water (100 mL, 10 mg/L) at with the same dosage (0.8 g). Then, after different treatment times, the removal of fluoride by the three materials was studied. The results are shown in Fig 5A. The fluoride removal efficiency of the original *Artemia* eggshell was very low, but the Aes-Z composite showed a relatively high defluorination efficiency. The removal efficiency of the Aes-Z composite reached 92.7% after 30 min. After treatment, the concentration of fluoride was 0.73 mg/L, which met the treatment requirements of the national standards (< 1.0 mg/L). Fig 5A also shows that the adsorption process of the composite reached equilibrium at 30 min, so the treatment time of the further studies was also 30 min.

#### Effect of adsorbent dosage

The removal percentages of fluoride with different dosages of adsorption materials are shown in Fig 5B. The figure also shows fluoride removal by zirconia and the Aes-Z composite. The operating pH of the solution was 6.0. For the solution containing 10 mg/L fluoride, the dosages of Aes-Z and zirconia have a positive correlation with the defluorination effect, and the concentration of fluoride reached the national standards (< 1.0 mg/L) at a dosage of 0.8 g.

For the *Artemia* Aes-Z composite, the removal percentage was only 30.5% by adding 0.1g of Aes-Z composite. When the dosage was 0.4 g, the removal percentage reached 81.3%. The removal percentage was 92.7% with 0.8 g of Aes-Z composite. The removal percentage increased to 93.1% with 1.0 g of Aes-Z composite.

#### Effect of pH

The adsorption of fluoride by Aes-Z adsorbent was discussed when the pH value of the solution ranged from 4 to 10. The results are shown in Fig 5C. With the change in pH value (4.0–10.00) the removal percentage of fluoride was still in the range of 90–93%.

#### Effect of fluoride concentration

The fluoride concentration of the solution in this study ranged from 2 to 40 mg/L. The results are shown in Fig 5D. The defluorination capacity of Aes-Z was improved with higher fluoride content in the solution.

#### Effect of other co-existing anions

**Fig 6:**
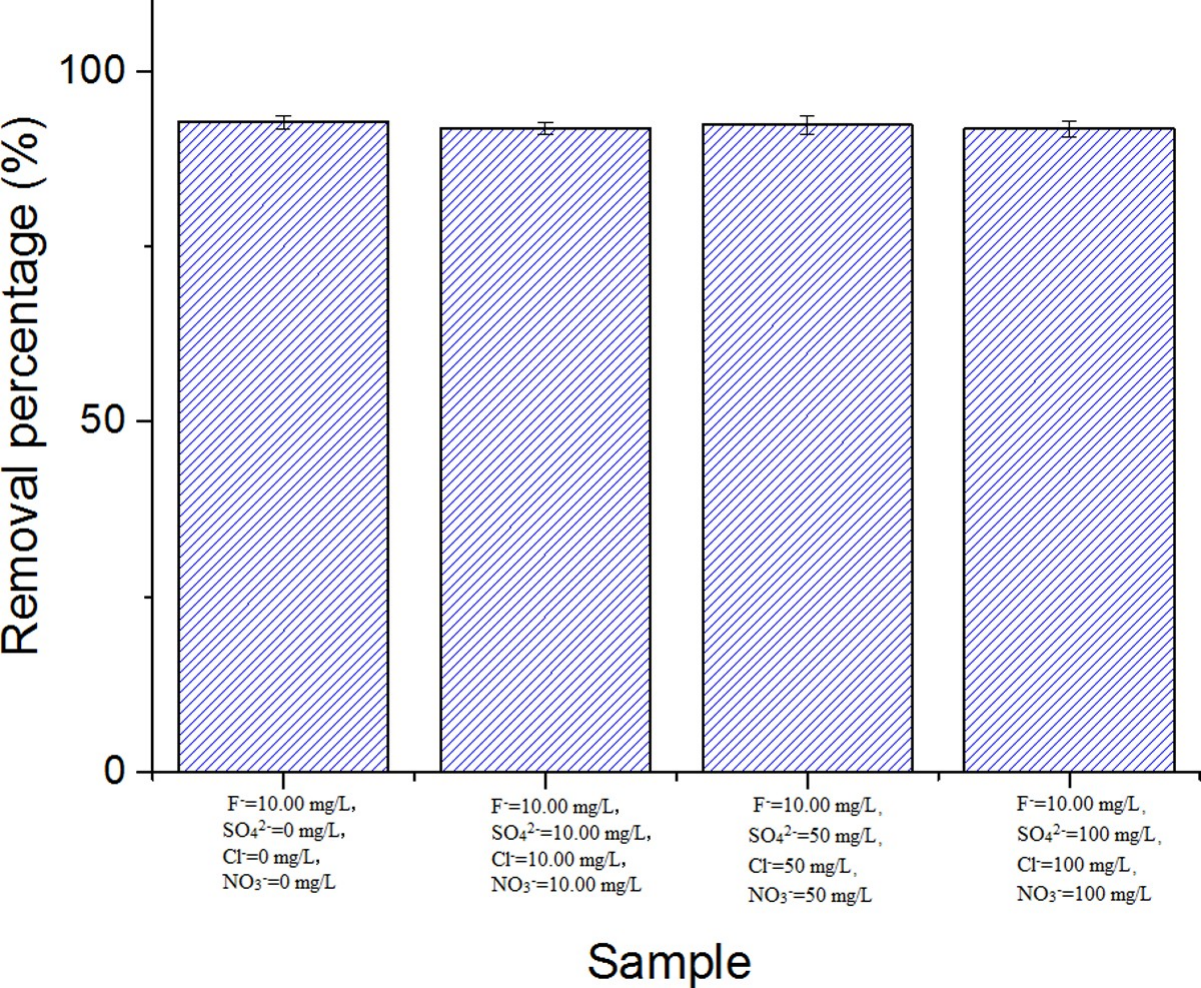
Effect of co-existing ion concentrations on fluoride removal efficiency.

The effect of other co-existing anions on defluorination is shown in Fig 6. In the concentration range (0–100 mg/L), the removal percentage of fluoride was stable.

#### Desorption and reuse of the adsorbent

The results show that the adsorption capacities of cycles 2, 3, and 4 were 95.24%, 91.35% and 88.74% that of cycle 1, respectively.

*Sorption kinetic models*. As in most studies, two models [7, 8] were selected to simulate the adsorption data in this research. The two models are as follows:

Pseudo-first-order rate model:

$$ln\left(q_{e}-q_{t}\right)=lnq_{e}-k_{1}t$$ (3)

Pseudo-second-order rate model:

$$t/q_{t}=\;1/k_{2}q_{e}{}^{2}+t/q_{e}$$ (4)

Here *k*_*1*_ (min^−1^) and *k*_*2*_ (g·mg^−1^·min^−1^) are the rate constants of pseudo-first-order and pseudo-second-order models respectively, meanwhile *q*_*e*_ (mg·g^−1^) and *q*_*t*_ (mg·g^−1^) are the adsorption capacities at equilibrium and any time *t*, respectively; and *t* is the reaction time (min). After calculating the corresponding values according to the models, *k*_*1*_ and *k*_*2*_ can be provided by the slope and intercept of the straight line after fitting.

**Fig 7:**
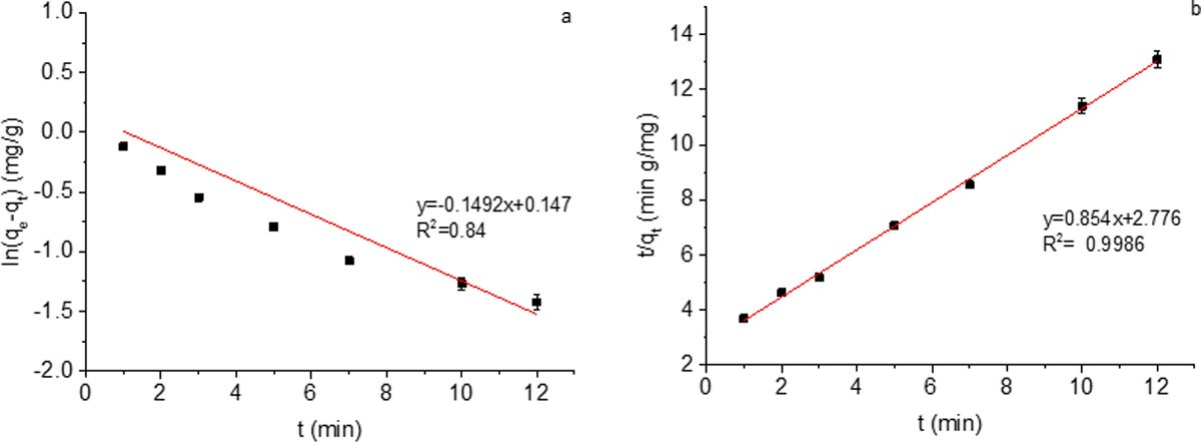
Test of (a) pseudo-first-order kinetic model and (b) pseudo-second-order kinetic model for the fluoride adsorption by *Artemia* eggshell-zirconium material.

As shown in Fig 7, the coefficient of determination (R^2^) of the pseudo-first-order rate kinetic model was only 0.84. For the pseudo-second-order kinetic model, R^2^ after fitting was 0.9986.

### Adsorption mechanism

**Fig 8:**
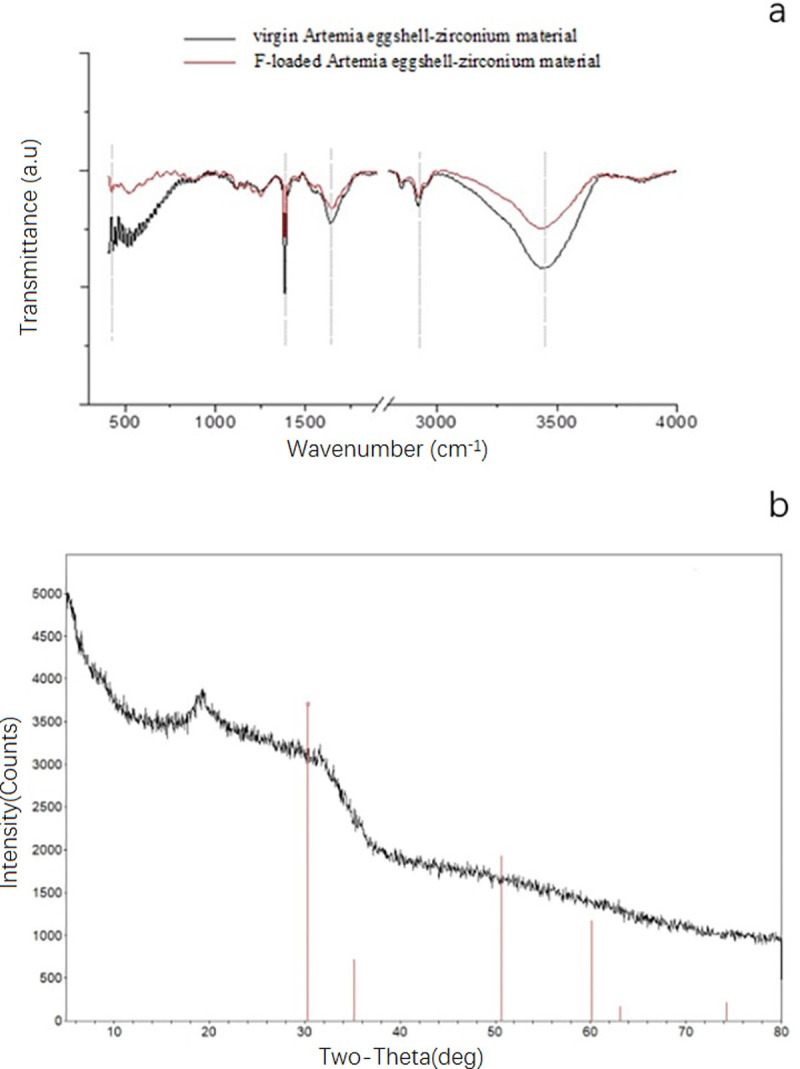
FTIR and XRD spectra of F-loaded *Artemia* eggshell-zirconium material. (a: FTIR spectra of the materials: Virgin *Artemia* eggshell-zirconium material and F-loaded *Artemia* eggshell-zirconium material; b: XRD pattern of F-loaded *Artemia* eggshell-zirconium material).

The FTIR spectra measurements of the composite material before and after adsorption are presented in Fig 8A. The intensity of the peak around 3453 cm^−1^ was obvious decreased after adsorption. The intensity of the peak at 2930 cm^−1^ changed little before and after adsorption. The peak around 1383 cm^−1^ also changed before and after adsorption. Fig 8B shows the XRD pattern of the F^−^ loaded sorbent. Compared with Fig 4, this figure shows that the reflections at 2θ-30.8° disappeared, but the peak at 2θ–20° remained.

## Discussion

According to the SEM results of the Aes-Z material, zirconia was successfully fixed to the inner surface of the eggshell. In addition, there was no pore blockage, and the pore size of the inner macropores was approximately 200–500 nm. Zirconia was fixed by coating and the Aes-Z material conserved a mechanism of large pore mass transfer effect. According to the TEM results, the eggshell played the role of a nano-template, and the maximum size of zirconia particles fixed to the inner surface of the eggshell pore was approximately 10–50 nm. The broad reflections at 2*θ*–30.8° of the Aes-Z composite were indexed to the (0 1 1) reflections of ZrO_2_, which indicating that ZrO_2_ nanoparticles were formed in the *Artemia* eggshell. Therefore, the *Artemia* eggshell has evident advantages over traditional carrier materials.

Combined with the results of the previous determination of zirconia content in the composite, the content of zirconia in the composite was lower than that of pure zirconia. However, the effect of defluorination was not affected, indicating that the composite is a promising defluorination material. With the extension of time, the adsorption of zirconia and Aes-Z composite on fluoride increased gradually.

The amount of adsorbent has an important influence on the adsorption efficiency [31]. When the dosage was more than 0.2 g, the defluorination effect of the Aes-Z composite material was better than that of zirconia. There were two possible reasons for this. On the one hand, the special pore structure of the *Artemia* shell provided excellent conditions for ion diffusion; on the other hand, the *Artemia* shell material realized the immobilization of inorganic particles through the nano-template effect, and the loaded nanozirconia fully played its adsorption role. The nano template effect formed by the *Artemia* eggshell makes the loaded zirconia form a nano state. The nanostructure increases the contact area between the material and F^-^ and significantly improves the adsorption capacity [32]. The removal percentage was 92.7% with 0.8 g of Aes-Z composite. However, the removal percentage increased to 93.1% with only 1.0 g of Aes-Z composite. At a fixed fluoride concentration, a higher dosage of material led to a the lower q_e_ value. Therefore, in the solutions with fixed fluoride concentrations (10 mg/L, 100 mL), 0.8 g was the best dosage.

The removal of fluoride by adsorption materials is affected by the pH value of the aqueous solution, as it affects the zeta potential of the materials, thus affecting the adsorption of fluoride [33]. When pH >7, the efficiency of defluorination decreased. The pH_ZPC_ (zero-charge pH value) of the composite material is between 6 and 7. Therefore, under alkaline conditions, the zeta potential of the material becomes negative, which may result in the repulsion of fluoride ions from the adsorbent.

Through the detection of drinking water sources in China, such as groundwater, the concentration of fluoride ion is usually not more than 15 mg/L. The fluoride content of groundwater can reach 35 mg/L worldwide [5]. However, the fluoride concentration must be controlled below 1.0 mg/L [4]. Therefore, the fluoride concentration in this study was changed from 2 to 40 mg/L. The results show that the defluorination capacity of Aes-Z improved with the higher fluoride content in the solution. This indicates that the zirconium-loaded biological composite adsorbent has a strong adsorption capacity and can be widely used in the defluorination of water.

There are some other anions in natural water that may interfere with the adsorption of fluoride. Therefore, in this study, multiple adsorption experiments were conducted to study the influence of competitive ions on fluoride removal. In our previous experiments, through the analysis of groundwater samples in the high fluoride area of groundwater in Northern China, the common anions in the water included Cl^-^, SO_4_^2-^, and NO_3_^-^, but their concentrations varied significantly at different sampling points, similar to the results reported in the literature [34]. The average concentrations of Cl^-^, SO_4_^2-^, and NO_3_^-^ in the groundwater samples were 86, 52, and 15 mg/L, respectively. In this study, water (neutral pH), containing all these anions and fluoride, was prepared and treated, unlike the methods used in other studies [8, 35]. The results are shown in Fig 6. In the concentration range (0–100 mg/L), other anions had little effect on the adsorption and removal of fluoride. When the concentration of sulfate ions was higher than 60 mg/L, the removal efficiency of fluoride decreased by 20% with other zirconia-loaded materials [8]. The composite material prepared in this study, showed evident advantages compared with other materials, namely an acceptable ability to bind fluorine ions, strong selectivity, and a stable performance. These may be related to the final structural characteristics of fluoride removal materials. It has been reported that the structural characteristics of the same adsorbent were different and the influence of other anions on the adsorption of fluoride ions are also different [36]. As reported in reference [24], the defluorination process of a special zirconia hybrid material was slightly affected by other anions. Our subsequent research will focus on the relationship between the structural characteristics and adsorption properties of these materials.

The reuse of materials can effectively reduce the cost of treatment. This is because, the material is negatively charged in alkaline conditions and repels fluoride ions; it can be desorbed and recovered under alkaline conditions. To determine the regeneration properties of the composite material, desorption and reuse experiments were developed. The results show that the adsorbent can be reused and remove fluoride effectively in four cycles.

The study of adsorption kinetics can not only predict the removal rate of pollutants in water, but can also provide a significant reference for the adsorption reaction mechanism research [37]. Two models were selected to simulate the adsorption data. The value of R^2^ is close to 1, indicating that the regression line has a better fitting degree to the observed value. If the value of R^2^ is small, it indicates that the regression line has a worse fitting degree to the observed value. For the pseudo-second-order kinetic model, R^2^ after fitting was 0.9986, indicating that the fluoride adsorption by Aes-Z conformed to the pseudo-second-order rate kinetic model. Meanwhile, the kinetic model reveals that the rate-limiting step in the fluoride removal process may be chemical adsorption; that is, there is electron sharing or exchange between F^-^ and the adsorption material in the adsorption process [38]. As the pore structure of *Artemia* eggshell could enhance the diffusion of F^-^, diffusion did not become a speed limiting factor. Further owing to the special nano-template effect of *Artemia* eggshell, it was conducive to the dispersion of zirconia. The fluoride ions and zirconia contacted sufficiently, so the ultimate speed limiting factor was chemical rather than physical.

The FTIR spectra measurements of the composite material before and after adsorption are presented in Fig 8. The peaks were around 3453 cm^−1^, which was caused by the stretching vibration of O-H. The slight movement of the peak and the evident decrease in the intensity after adsorption indicate that OH may participate in the fluoride adsorption. The C-H stretching vibration peak was around 2930 cm^−1^. The intensity of the peak at 2930 cm^−1^ changed little before and after adsorption, indicating that the molecular structure of the biomass carrier should be unchanged. The peak around 1643 cm^−1^ was due to the bending vibration of water molecules. This indicates the presence of surface-adsorbed water. The peak around 1383 cm^−1^ was caused by the C-H bending vibration. The peak also changed little before and after adsorption. Meanwhile, the Zr-F stretching vibration peak is always approximately 375–475 cm^−1^. This peak also appeared in the spectrum of the adsorbed material. For the XRD analysis, the characteristic peak of zirconia disappeared, indicating that, after adsorption, the crystalline structure was different. However, the peak at 2θ–20° still appeared, indicating that the eggshell provided the diffusion channel for the fluoride ions but was not responsible for the adsorption.

**Fig 9:**
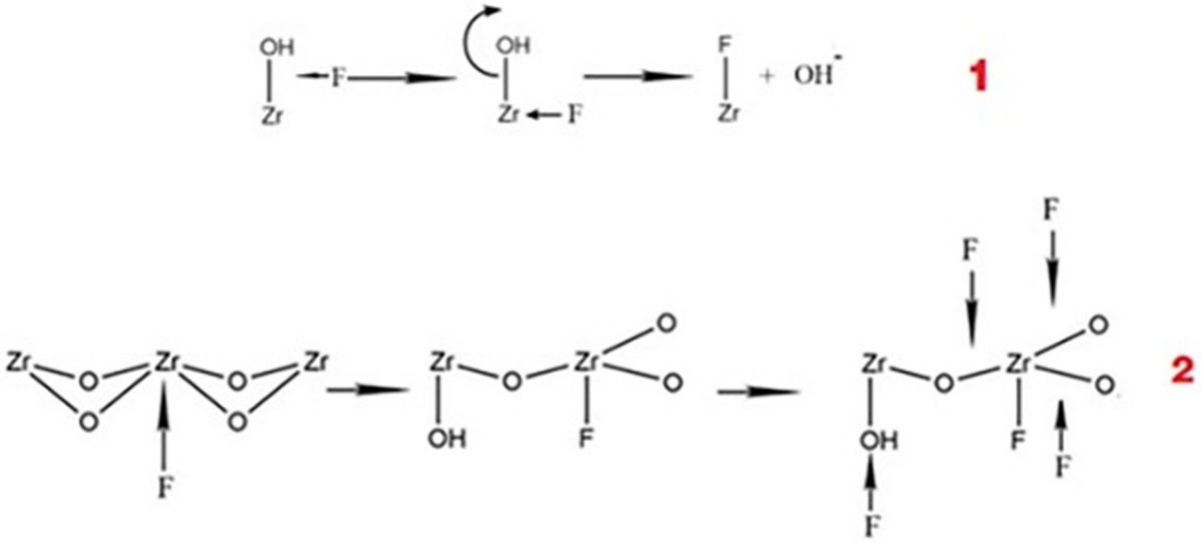
Inferred mechanisms of fluoride adsorption by *Artemia* eggshell-zirconium material.

Combined with the experimental results of the FTIR, XRD, adsorption kinetics and zeta potential analyses, the inferred adsorption reaction of the fluoride and Aes-Z material was proposed (Fig 9). There are two types of reactions; one is similar to the reaction reported in the literature [8]. Zirconium dioxide is an amphoteric metal compound with active hydroxyl groups on the metal surface [39]; owing to the high electronegativity of F, F ions replace the hydroxyl groups (Fig 9–1). In the second type of reaction, owing to the strong affinity of zirconium to fluoride ions, the continuous proximity of fluoride ions causes the chemical bond between zirconium and oxygen to break, and the residual oxygen combines with hydrogen to form a hydroxyl group (Fig 9–2). Then, fluoride ions continue to combine with zirconium to replace the hydroxyl group and finally form coordination compounds, similar to the reaction reported in the reference [40]. According to the speculated mechanism, the pH value of the solution should increase after the adsorption reaction; this was also confirmed via experiments.

## Conclusions

Fluoride pollution in drinking water has attracted increasing attention worldwide. The development of new adsorption materials helps solve the problem of fluoride concentration over-standards in drinking water. A new Aes-Z composite material was prepared, showing a suitable performance for fluoride adsorption. The removal efficiency of fluoride reached 93% at 30 min with an initial fluoride concentration of 10 mg/L. Furthermore, the adsorption of fluoride was not affected by pH (4–10) and common anions (Cl^-^, SO_4_^2-^, and NO_3_^-^) in water. The defluorination effect of the composite material was better than that of zirconia. The kinetic model revealed that the rate-limiting step in the removal process was chemical adsorption. Owing to the special nano-template effect of *Artemia* eggshell, it was conducive to the dispersion of zirconia and the fluoride ions and zirconia experienced sufficient contact. This study demonstrates that Aes-Z is a promising adsorbent material for fluoride removal.

## Supporting information

S1 File (DOCX)
