# Supplementary material for: Development of a novel Artemia eggshell-zirconium nanocomposite for efficient fluoride removal
Source: PLoS One. 2021 Jan 4;16(1):e0244711. doi: 10.1371/journal.pone.0244711 (PMC7781666; doi:10.1371/journal.pone.0244711)
Supplement: S1 File — (DOCX) [file pone.0244711.s001.docx]

Figure 5 a

mean±SD

| Time | removal | SD | removal | SD | removal | SD |
| --- | --- | --- | --- | --- | --- | --- |
| 0 | 0 | 0 | 0 | 0 | 0 | 0 |
| 0.25 | 0 | 0 | 87.79 | 1.25 | 86.91 | 0.78 |
| 0.5 | 0 | 0 | 90.55 | 0.46 | 92.7 | 0.98 |
| 1 | 0 | 0 | 92.11 | 0.33 | 92.32 | 1.02 |
| 3 | 0 | 0 | 92.82 | 1.12 | 93.88 | 1.44 |
| 6 | 0 | 0 | 92.95 | 1.48 | 94.58 | 1.59 |
| 9 | 0 | 0 | 93.41 | 2.04 | 94.82 | 2.05 |
| 12 | 2.65 | 0.43 | 93.49 | 1.15 | 94.72 | 2.11 |
| 24 | 5 | 1.26 | 92.5 | 1.96 | 94.73 | 2.25 |

Figure 5 b

mean±SD

| dosage | removal | SD | removal | SD |
| --- | --- | --- | --- | --- |
| 0.1 | 30.52 | 1.24 | 40.26 | 0.48 |
| 0.2 | 52.06 | 1.56 | 61.52 | 0.56 |
| 0.4 | 81.3 | 2.14 | 80.12 | 1.22 |
| 0.6 | 89.86 | 2.02 | 86.82 | 1.45 |
| 0.8 | 92.7 | 0.98 | 90.55 | 0.46 |
| 1 | 93.7 | 1.02 | 92.2 | 1.25 |

Figure 5 c

mean±SD

| pH | removal | SD |
| --- | --- | --- |
| 4 | 92.78 | 1.25 |
| 5 | 93.01 | 1.22 |
| 6 | 92.7 | 0.98 |
| 7 | 92.51 | 0.45 |
| 8 | 91.7 | 1.22 |
| 9 | 91.43 | 0.85 |
| 10 | 90.28 | 1.52 |

Figure 5 d

mean±SD

| concentration | removal | SD | capacity | SD |
| --- | --- | --- | --- | --- |
| 2 | 72.69 | 0.88 | 0.1817 | 0.08 |
| 5 | 83.75 | 0.87 | 0.5234 | 0.09 |
| 10 | 92.7 | 0.98 | 1.159 | 0.07 |
| 20 | 80.4 | 1.02 | 2.01 | 0.07 |
| 30 | 70.2 | 1.03 | 2.63 | 0.08 |
| 40 | 67.86 | 1.05 | 3.39 | 0.07 |

Figure 6

mean±SD

|  | removal | SD |
| --- | --- | --- |
| 1 | 92.7 | 0.98 |
| 2 | 91.75 | 0.87 |
| 3 | 92.3 | 1.28 |
| 4 | 92 | 1.08 |

Figure 7

mean±SD

| Time（min） | 0 | 1 | 2 | 3 | 5 | 7 | 10 | 12 |
| --- | --- | --- | --- | --- | --- | --- | --- | --- |
| ln(qe-qt) | 0.147 | -0.1187 | -0.3188 | -0.54576 | -0.794 | -1.07587 | -1.268 | -1.4217 |
| SD | - | 0 | 0.0026 | 0.0021 | 0.02 | 0.02 | 0.05 | 0.06 |
| t/q_t_ | - | 3.69 | 4.63 | 5.176 | 7.074 | 8.55 | 11.395 | 13.076 |
| SD | - | 0.01 | 0.02 | 0.01 | 0.1 | 0.08 | 0.28 | 0.305 |

Figure 8a

Infrared spectrum data

| Wavenumber | Virgin | F-loaded |
| --- | --- | --- |
| 3996.452 | 0.99843 | 0.99942 |
| 3994.524 | 0.99843 | 0.99942 |
| 3992.595 | 0.99843 | 0.99942 |
| 3990.666 | 0.99843 | 0.99942 |
| 3988.737 | 0.99843 | 0.99942 |
| 3986.809 | 0.99837 | 0.99942 |
| 3984.88 | 0.99826 | 0.99942 |
| 3982.951 | 0.99817 | 0.99924 |
| 3981.022 | 0.99815 | 0.99905 |
| 3979.093 | 0.99822 | 0.99889 |
| 3977.165 | 0.99837 | 0.99879 |
| 3975.236 | 0.99857 | 0.99874 |
| 3973.307 | 0.99868 | 0.99865 |
| 3971.378 | 0.99861 | 0.99855 |
| 3969.449 | 0.99837 | 0.99853 |
| 3967.521 | 0.99799 | 0.99857 |
| 3965.592 | 0.99756 | 0.99865 |
| 3963.663 | 0.99719 | 0.99874 |
| 3961.734 | 0.997 | 0.99876 |
| 3959.805 | 0.99699 | 0.99873 |
| 3957.877 | 0.99709 | 0.99877 |
| 3955.948 | 0.9972 | 0.99881 |
| 3954.019 | 0.99724 | 0.99867 |
| 3952.09 | 0.99716 | 0.99843 |
| 3950.162 | 0.99691 | 0.99825 |
| 3948.233 | 0.99655 | 0.99818 |
| 3946.304 | 0.99619 | 0.99815 |
| 3944.375 | 0.99592 | 0.99791 |
| 3942.446 | 0.9958 | 0.99748 |
| 3940.518 | 0.99584 | 0.99714 |
| 3938.589 | 0.99603 | 0.99692 |
| 3936.66 | 0.99627 | 0.99667 |
| 3934.731 | 0.9964 | 0.9965 |
| 3932.802 | 0.99632 | 0.99645 |
| 3930.874 | 0.99605 | 0.99636 |
| 3928.945 | 0.99569 | 0.99623 |
| 3927.016 | 0.99529 | 0.99623 |
| 3925.087 | 0.99499 | 0.99641 |
| 3923.158 | 0.9949 | 0.99656 |
| 3921.23 | 0.99498 | 0.99654 |
| 3919.301 | 0.99511 | 0.99661 |
| 3917.372 | 0.99514 | 0.99681 |
| 3915.443 | 0.99501 | 0.99677 |
| 3913.515 | 0.99472 | 0.99642 |
| 3911.586 | 0.99431 | 0.99609 |
| 3909.657 | 0.99387 | 0.99603 |
| 3907.728 | 0.99347 | 0.99629 |
| 3905.799 | 0.99325 | 0.99658 |
| 3903.871 | 0.99325 | 0.99653 |
| 3901.942 | 0.99341 | 0.99635 |
| 3900.013 | 0.99369 | 0.99642 |
| 3898.084 | 0.99395 | 0.99647 |
| 3896.155 | 0.99404 | 0.99625 |
| 3894.227 | 0.99382 | 0.99581 |
| 3892.298 | 0.9933 | 0.99502 |
| 3890.369 | 0.99269 | 0.99414 |
| 3888.44 | 0.99215 | 0.99367 |
| 3886.511 | 0.99167 | 0.99366 |
| 3884.583 | 0.99134 | 0.99394 |
| 3882.654 | 0.99126 | 0.9939 |
| 3880.725 | 0.99137 | 0.99329 |
| 3878.796 | 0.99153 | 0.99304 |
| 3876.867 | 0.9917 | 0.99331 |
| 3874.939 | 0.99191 | 0.99314 |
| 3873.01 | 0.99214 | 0.99266 |
| 3871.081 | 0.99209 | 0.99271 |
| 3869.152 | 0.99163 | 0.99334 |
| 3867.224 | 0.99095 | 0.99398 |
| 3865.295 | 0.99027 | 0.99367 |
| 3863.366 | 0.98973 | 0.99261 |
| 3861.437 | 0.98955 | 0.99213 |
| 3859.508 | 0.98986 | 0.99235 |
| 3857.58 | 0.9905 | 0.99266 |
| 3855.651 | 0.99109 | 0.99296 |
| 3853.722 | 0.9913 | 0.99306 |
| 3851.793 | 0.99125 | 0.99289 |
| 3849.864 | 0.99104 | 0.9927 |
| 3847.936 | 0.99062 | 0.99285 |
| 3846.007 | 0.99006 | 0.99333 |
| 3844.078 | 0.98963 | 0.99319 |
| 3842.149 | 0.98957 | 0.99196 |
| 3840.22 | 0.98989 | 0.99099 |
| 3838.292 | 0.99044 | 0.9913 |
| 3836.363 | 0.99111 | 0.99217 |
| 3834.434 | 0.9917 | 0.99257 |
| 3832.505 | 0.99177 | 0.99232 |
| 3830.577 | 0.9913 | 0.99229 |
| 3828.648 | 0.99072 | 0.99252 |
| 3826.719 | 0.99047 | 0.9922 |
| 3824.79 | 0.99059 | 0.99195 |
| 3822.861 | 0.99091 | 0.9925 |
| 3820.933 | 0.99147 | 0.99317 |
| 3819.004 | 0.99216 | 0.9933 |
| 3817.075 | 0.9926 | 0.99338 |
| 3815.146 | 0.99262 | 0.99378 |
| 3813.217 | 0.99242 | 0.99394 |
| 3811.289 | 0.99217 | 0.9936 |
| 3809.36 | 0.99189 | 0.99334 |
| 3807.431 | 0.99175 | 0.99358 |
| 3805.502 | 0.99203 | 0.99384 |
| 3803.573 | 0.99269 | 0.99384 |
| 3801.645 | 0.99342 | 0.99414 |
| 3799.716 | 0.99406 | 0.99471 |
| 3797.787 | 0.99457 | 0.99483 |
| 3795.858 | 0.99473 | 0.99451 |
| 3793.93 | 0.99443 | 0.99446 |
| 3792.001 | 0.99391 | 0.99445 |
| 3790.072 | 0.99347 | 0.99389 |
| 3788.143 | 0.99323 | 0.99346 |
| 3786.214 | 0.99319 | 0.99374 |
| 3784.286 | 0.99342 | 0.99415 |
| 3782.357 | 0.99389 | 0.99432 |
| 3780.428 | 0.99437 | 0.99431 |
| 3778.499 | 0.99478 | 0.99425 |
| 3776.57 | 0.99507 | 0.9942 |
| 3774.642 | 0.99515 | 0.99429 |
| 3772.713 | 0.99502 | 0.99444 |
| 3770.784 | 0.99485 | 0.99432 |
| 3768.855 | 0.99468 | 0.99406 |
| 3766.926 | 0.9945 | 0.99423 |
| 3764.998 | 0.99443 | 0.99473 |
| 3763.069 | 0.99459 | 0.9947 |
| 3761.14 | 0.99476 | 0.99425 |
| 3759.211 | 0.99477 | 0.99427 |
| 3757.283 | 0.99482 | 0.9944 |
| 3755.354 | 0.9951 | 0.99387 |
| 3753.425 | 0.99542 | 0.99354 |
| 3751.496 | 0.99556 | 0.99423 |
| 3749.567 | 0.99567 | 0.99496 |
| 3747.639 | 0.99584 | 0.99488 |
| 3745.71 | 0.9959 | 0.99461 |
| 3743.781 | 0.99586 | 0.99471 |
| 3741.852 | 0.99601 | 0.99476 |
| 3739.923 | 0.99633 | 0.99443 |
| 3737.995 | 0.99642 | 0.9943 |
| 3736.066 | 0.99617 | 0.99459 |
| 3734.137 | 0.99586 | 0.99436 |
| 3732.208 | 0.99566 | 0.99347 |
| 3730.279 | 0.99549 | 0.99339 |
| 3728.351 | 0.99527 | 0.99441 |
| 3726.422 | 0.99511 | 0.9953 |
| 3724.493 | 0.99507 | 0.99527 |
| 3722.564 | 0.99515 | 0.99463 |
| 3720.636 | 0.99545 | 0.99434 |
| 3718.707 | 0.99597 | 0.9949 |
| 3716.778 | 0.99656 | 0.99584 |
| 3714.849 | 0.99694 | 0.9963 |
| 3712.92 | 0.99689 | 0.99609 |
| 3710.992 | 0.99657 | 0.99574 |
| 3709.063 | 0.99635 | 0.99558 |
| 3707.134 | 0.99635 | 0.99578 |
| 3705.205 | 0.99637 | 0.99641 |
| 3703.276 | 0.99629 | 0.99692 |
| 3701.348 | 0.99619 | 0.99642 |
| 3699.419 | 0.99614 | 0.99529 |
| 3697.49 | 0.99611 | 0.99488 |
| 3695.561 | 0.99615 | 0.99549 |
| 3693.632 | 0.99615 | 0.99627 |
| 3691.704 | 0.99586 | 0.99668 |
| 3689.775 | 0.99529 | 0.99677 |
| 3687.846 | 0.99473 | 0.99651 |
| 3685.917 | 0.99439 | 0.99629 |
| 3683.989 | 0.99432 | 0.99675 |
| 3682.06 | 0.99444 | 0.9972 |
| 3680.131 | 0.99467 | 0.99688 |
| 3678.202 | 0.99485 | 0.99631 |
| 3676.273 | 0.99486 | 0.9961 |
| 3674.345 | 0.99473 | 0.99632 |
| 3672.416 | 0.9944 | 0.99672 |
| 3670.487 | 0.99374 | 0.9969 |
| 3668.558 | 0.99283 | 0.9966 |
| 3666.629 | 0.99185 | 0.99595 |
| 3664.701 | 0.99087 | 0.99538 |
| 3662.772 | 0.98997 | 0.99511 |
| 3660.843 | 0.98935 | 0.99468 |
| 3658.914 | 0.98903 | 0.99371 |
| 3656.985 | 0.98884 | 0.99313 |
| 3655.057 | 0.98867 | 0.99357 |
| 3653.128 | 0.98843 | 0.99416 |
| 3651.199 | 0.98799 | 0.9943 |
| 3649.27 | 0.98728 | 0.99416 |
| 3647.342 | 0.98645 | 0.99375 |
| 3645.413 | 0.98554 | 0.99323 |
| 3643.484 | 0.98444 | 0.99312 |
| 3641.555 | 0.98322 | 0.99287 |
| 3639.626 | 0.9821 | 0.99145 |
| 3637.698 | 0.98115 | 0.98977 |
| 3635.769 | 0.98028 | 0.98934 |
| 3633.84 | 0.97952 | 0.98992 |
| 3631.911 | 0.97893 | 0.99024 |
| 3629.982 | 0.97825 | 0.98985 |
| 3628.054 | 0.97744 | 0.98942 |
| 3626.125 | 0.97666 | 0.98928 |
| 3624.196 | 0.97598 | 0.98923 |
| 3622.267 | 0.97525 | 0.98919 |
| 3620.338 | 0.97438 | 0.98873 |
| 3618.41 | 0.97346 | 0.98747 |
| 3616.481 | 0.97251 | 0.98624 |
| 3614.552 | 0.97148 | 0.98596 |
| 3612.623 | 0.97035 | 0.98604 |
| 3610.695 | 0.96915 | 0.98564 |
| 3608.766 | 0.96792 | 0.98468 |
| 3606.837 | 0.96676 | 0.98355 |
| 3604.908 | 0.96582 | 0.98288 |
| 3602.979 | 0.96509 | 0.98274 |
| 3601.051 | 0.96448 | 0.98251 |
| 3599.122 | 0.96387 | 0.9817 |
| 3597.193 | 0.96314 | 0.98073 |
| 3595.264 | 0.96224 | 0.98035 |
| 3593.335 | 0.96127 | 0.98046 |
| 3591.407 | 0.96026 | 0.98041 |
| 3589.478 | 0.95916 | 0.97997 |
| 3587.549 | 0.95799 | 0.9794 |
| 3585.62 | 0.95694 | 0.97876 |
| 3583.691 | 0.956 | 0.97819 |
| 3581.763 | 0.95505 | 0.97788 |
| 3579.834 | 0.95405 | 0.97745 |
| 3577.905 | 0.95306 | 0.9764 |
| 3575.976 | 0.95211 | 0.97524 |
| 3574.047 | 0.95123 | 0.97476 |
| 3572.119 | 0.95055 | 0.97471 |
| 3570.19 | 0.95005 | 0.97449 |
| 3568.261 | 0.94957 | 0.97407 |
| 3566.332 | 0.94903 | 0.97355 |
| 3564.404 | 0.94846 | 0.97289 |
| 3562.475 | 0.94779 | 0.97229 |
| 3560.546 | 0.94689 | 0.97191 |
| 3558.617 | 0.94579 | 0.97138 |
| 3556.688 | 0.94461 | 0.97036 |
| 3554.76 | 0.94343 | 0.96941 |
| 3552.831 | 0.94232 | 0.96908 |
| 3550.902 | 0.94144 | 0.96899 |
| 3548.973 | 0.94083 | 0.96865 |
| 3547.044 | 0.94035 | 0.96822 |
| 3545.116 | 0.93985 | 0.96789 |
| 3543.187 | 0.93936 | 0.96755 |
| 3541.258 | 0.93882 | 0.96703 |
| 3539.329 | 0.93812 | 0.96662 |
| 3537.4 | 0.93725 | 0.9663 |
| 3535.472 | 0.93632 | 0.96588 |
| 3533.543 | 0.93541 | 0.96549 |
| 3531.614 | 0.9346 | 0.96523 |
| 3529.685 | 0.93395 | 0.965 |
| 3527.757 | 0.93349 | 0.96465 |
| 3525.828 | 0.93315 | 0.96415 |
| 3523.899 | 0.93283 | 0.96361 |
| 3521.97 | 0.93242 | 0.96314 |
| 3520.041 | 0.93185 | 0.96265 |
| 3518.113 | 0.93106 | 0.96202 |
| 3516.184 | 0.93009 | 0.96146 |
| 3514.255 | 0.92908 | 0.96105 |
| 3512.326 | 0.92817 | 0.96074 |
| 3510.397 | 0.92746 | 0.96038 |
| 3508.469 | 0.92702 | 0.95997 |
| 3506.54 | 0.9268 | 0.95961 |
| 3504.611 | 0.92666 | 0.95927 |
| 3502.682 | 0.92646 | 0.95886 |
| 3500.753 | 0.92611 | 0.95841 |
| 3498.825 | 0.92553 | 0.958 |
| 3496.896 | 0.9247 | 0.95759 |
| 3494.967 | 0.92373 | 0.95713 |
| 3493.038 | 0.92281 | 0.9568 |
| 3491.11 | 0.92204 | 0.95662 |
| 3489.181 | 0.92151 | 0.95635 |
| 3487.252 | 0.92121 | 0.95599 |
| 3485.323 | 0.9211 | 0.95572 |
| 3483.394 | 0.921 | 0.95549 |
| 3481.466 | 0.92076 | 0.95518 |
| 3479.537 | 0.92035 | 0.95479 |
| 3477.608 | 0.91978 | 0.95443 |
| 3475.679 | 0.91909 | 0.95411 |
| 3473.75 | 0.91841 | 0.9538 |
| 3471.822 | 0.91788 | 0.95351 |
| 3469.893 | 0.91757 | 0.95323 |
| 3467.964 | 0.91749 | 0.95291 |
| 3466.035 | 0.91759 | 0.95257 |
| 3464.106 | 0.91776 | 0.95227 |
| 3462.178 | 0.91784 | 0.95198 |
| 3460.249 | 0.91775 | 0.9517 |
| 3458.32 | 0.91746 | 0.95148 |
| 3456.391 | 0.91701 | 0.95132 |
| 3454.463 | 0.91649 | 0.95117 |
| 3452.534 | 0.91604 | 0.95105 |
| 3450.605 | 0.91578 | 0.95092 |
| 3448.676 | 0.91574 | 0.95076 |
| 3446.747 | 0.91592 | 0.95058 |
| 3444.819 | 0.91626 | 0.95043 |
| 3442.89 | 0.91664 | 0.95034 |
| 3440.961 | 0.91691 | 0.95027 |
| 3439.032 | 0.91701 | 0.95021 |
| 3437.103 | 0.91692 | 0.95022 |
| 3435.175 | 0.91671 | 0.95031 |
| 3433.246 | 0.9165 | 0.95037 |
| 3431.317 | 0.91641 | 0.95039 |
| 3429.388 | 0.91655 | 0.95041 |
| 3427.459 | 0.91693 | 0.95041 |
| 3425.531 | 0.91747 | 0.95045 |
| 3423.602 | 0.91808 | 0.95055 |
| 3421.673 | 0.91861 | 0.95069 |
| 3419.744 | 0.91897 | 0.95085 |
| 3417.816 | 0.91914 | 0.95103 |
| 3415.887 | 0.91918 | 0.95127 |
| 3413.958 | 0.9192 | 0.95156 |
| 3412.029 | 0.91931 | 0.95188 |
| 3410.1 | 0.91961 | 0.95222 |
| 3408.172 | 0.9201 | 0.95253 |
| 3406.243 | 0.92074 | 0.95276 |
| 3404.314 | 0.92141 | 0.953 |
| 3402.385 | 0.92202 | 0.95329 |
| 3400.456 | 0.92249 | 0.95356 |
| 3398.528 | 0.92279 | 0.95378 |
| 3396.599 | 0.92299 | 0.95401 |
| 3394.67 | 0.92319 | 0.95426 |
| 3392.741 | 0.92352 | 0.95451 |
| 3390.812 | 0.92404 | 0.95475 |
| 3388.884 | 0.92476 | 0.955 |
| 3386.955 | 0.92562 | 0.95531 |
| 3385.026 | 0.92653 | 0.95563 |
| 3383.097 | 0.92737 | 0.95599 |
| 3381.169 | 0.92807 | 0.95641 |
| 3379.24 | 0.92863 | 0.95687 |
| 3377.311 | 0.92909 | 0.95734 |
| 3375.382 | 0.92951 | 0.95786 |
| 3373.453 | 0.93 | 0.95843 |
| 3371.525 | 0.93062 | 0.95899 |
| 3369.596 | 0.9314 | 0.9595 |
| 3367.667 | 0.93232 | 0.96 |
| 3365.738 | 0.93329 | 0.96053 |
| 3363.809 | 0.93424 | 0.96109 |
| 3361.881 | 0.93508 | 0.96167 |
| 3359.952 | 0.93578 | 0.96222 |
| 3358.023 | 0.93636 | 0.96274 |
| 3356.094 | 0.93688 | 0.96326 |
| 3354.165 | 0.93743 | 0.96379 |
| 3352.237 | 0.93807 | 0.96432 |
| 3350.308 | 0.93883 | 0.96479 |
| 3348.379 | 0.9397 | 0.96522 |
| 3346.45 | 0.94063 | 0.96563 |
| 3344.522 | 0.94154 | 0.96604 |
| 3342.593 | 0.94235 | 0.96643 |
| 3340.664 | 0.94303 | 0.96678 |
| 3338.735 | 0.94358 | 0.9671 |
| 3336.806 | 0.94405 | 0.96739 |
| 3334.878 | 0.94454 | 0.9677 |
| 3332.949 | 0.94514 | 0.96806 |
| 3331.02 | 0.94588 | 0.96844 |
| 3329.091 | 0.94673 | 0.96882 |
| 3327.162 | 0.94762 | 0.96921 |
| 3325.234 | 0.94844 | 0.96964 |
| 3323.305 | 0.94912 | 0.97011 |
| 3321.376 | 0.94961 | 0.97056 |
| 3319.447 | 0.94993 | 0.971 |
| 3317.518 | 0.95014 | 0.97142 |
| 3315.59 | 0.95033 | 0.97183 |
| 3313.661 | 0.95063 | 0.97227 |
| 3311.732 | 0.95111 | 0.97268 |
| 3309.803 | 0.95179 | 0.97302 |
| 3307.875 | 0.95261 | 0.9733 |
| 3305.946 | 0.95344 | 0.97356 |
| 3304.017 | 0.95414 | 0.97386 |
| 3302.088 | 0.95463 | 0.97417 |
| 3300.159 | 0.95491 | 0.97444 |
| 3298.231 | 0.95505 | 0.97464 |
| 3296.302 | 0.95516 | 0.9748 |
| 3294.373 | 0.95534 | 0.97494 |
| 3292.444 | 0.95566 | 0.97508 |
| 3290.515 | 0.95614 | 0.9752 |
| 3288.587 | 0.95673 | 0.9753 |
| 3286.658 | 0.95736 | 0.97541 |
| 3284.729 | 0.95791 | 0.97556 |
| 3282.8 | 0.95828 | 0.97576 |
| 3280.871 | 0.95846 | 0.97601 |
| 3278.943 | 0.95848 | 0.97628 |
| 3277.014 | 0.95846 | 0.97652 |
| 3275.085 | 0.95851 | 0.97674 |
| 3273.156 | 0.95875 | 0.97696 |
| 3271.227 | 0.95921 | 0.97718 |
| 3269.299 | 0.95985 | 0.97739 |
| 3267.37 | 0.96057 | 0.97761 |
| 3265.441 | 0.96124 | 0.97787 |
| 3263.512 | 0.96176 | 0.97818 |
| 3261.584 | 0.96207 | 0.97851 |
| 3259.655 | 0.96217 | 0.97885 |
| 3257.726 | 0.96216 | 0.9792 |
| 3255.797 | 0.96215 | 0.97954 |
| 3253.868 | 0.96227 | 0.97984 |
| 3251.94 | 0.96262 | 0.98011 |
| 3250.011 | 0.9632 | 0.98036 |
| 3248.082 | 0.96395 | 0.98058 |
| 3246.153 | 0.96472 | 0.98076 |
| 3244.224 | 0.96536 | 0.98094 |
| 3242.296 | 0.96577 | 0.98115 |
| 3240.367 | 0.96594 | 0.98139 |
| 3238.438 | 0.96593 | 0.98161 |
| 3236.509 | 0.96586 | 0.98181 |
| 3234.58 | 0.96589 | 0.98201 |
| 3232.652 | 0.96612 | 0.98222 |
| 3230.723 | 0.96659 | 0.98243 |
| 3228.794 | 0.96724 | 0.9826 |
| 3226.865 | 0.96799 | 0.98276 |
| 3224.937 | 0.9687 | 0.98294 |
| 3223.008 | 0.96925 | 0.98318 |
| 3221.079 | 0.96961 | 0.98349 |
| 3219.15 | 0.96981 | 0.98383 |
| 3217.221 | 0.96992 | 0.98416 |
| 3215.293 | 0.97007 | 0.98449 |
| 3213.364 | 0.97033 | 0.98481 |
| 3211.435 | 0.97074 | 0.98512 |
| 3209.506 | 0.97128 | 0.98543 |
| 3207.577 | 0.97186 | 0.98568 |
| 3205.649 | 0.97239 | 0.98584 |
| 3203.72 | 0.97281 | 0.98597 |
| 3201.791 | 0.97311 | 0.98615 |
| 3199.862 | 0.97332 | 0.98635 |
| 3197.933 | 0.97351 | 0.98653 |
| 3196.005 | 0.97377 | 0.98669 |
| 3194.076 | 0.97415 | 0.98687 |
| 3192.147 | 0.97466 | 0.98708 |
| 3190.218 | 0.97525 | 0.98731 |
| 3188.29 | 0.97587 | 0.98754 |
| 3186.361 | 0.97642 | 0.98773 |
| 3184.432 | 0.97685 | 0.98793 |
| 3182.503 | 0.97713 | 0.98816 |
| 3180.574 | 0.97728 | 0.98844 |
| 3178.646 | 0.97739 | 0.98874 |
| 3176.717 | 0.97752 | 0.98901 |
| 3174.788 | 0.97777 | 0.98925 |
| 3172.859 | 0.97817 | 0.98949 |
| 3170.93 | 0.9787 | 0.98973 |
| 3169.002 | 0.97931 | 0.98995 |
| 3167.073 | 0.9799 | 0.99015 |
| 3165.144 | 0.98036 | 0.99033 |
| 3163.215 | 0.98062 | 0.99051 |
| 3161.286 | 0.9807 | 0.99073 |
| 3159.358 | 0.98067 | 0.99097 |
| 3157.429 | 0.98064 | 0.99117 |
| 3155.5 | 0.98073 | 0.99135 |
| 3153.571 | 0.98101 | 0.99152 |
| 3151.643 | 0.98148 | 0.99168 |
| 3149.714 | 0.98207 | 0.99184 |
| 3147.785 | 0.98268 | 0.99198 |
| 3145.856 | 0.98321 | 0.99212 |
| 3143.927 | 0.98357 | 0.99228 |
| 3141.999 | 0.98374 | 0.99246 |
| 3140.07 | 0.98378 | 0.99263 |
| 3138.141 | 0.98378 | 0.99279 |
| 3136.212 | 0.98385 | 0.99294 |
| 3134.283 | 0.98406 | 0.99307 |
| 3132.355 | 0.98442 | 0.99322 |
| 3130.426 | 0.98487 | 0.99337 |
| 3128.497 | 0.98529 | 0.9935 |
| 3126.568 | 0.98557 | 0.99358 |
| 3124.639 | 0.98568 | 0.99364 |
| 3122.711 | 0.98565 | 0.99371 |
| 3120.782 | 0.98556 | 0.9938 |
| 3118.853 | 0.98551 | 0.99388 |
| 3116.924 | 0.98561 | 0.99392 |
| 3114.996 | 0.9859 | 0.99395 |
| 3113.067 | 0.98636 | 0.99401 |
| 3111.138 | 0.98692 | 0.99409 |
| 3109.209 | 0.98749 | 0.99419 |
| 3107.28 | 0.98795 | 0.99432 |
| 3105.352 | 0.98826 | 0.99445 |
| 3103.423 | 0.98841 | 0.9946 |
| 3101.494 | 0.98846 | 0.99479 |
| 3099.565 | 0.98852 | 0.99502 |
| 3097.636 | 0.98868 | 0.99529 |
| 3095.708 | 0.98898 | 0.99555 |
| 3093.779 | 0.98942 | 0.9958 |
| 3091.85 | 0.98993 | 0.99604 |
| 3089.921 | 0.99043 | 0.99628 |
| 3087.992 | 0.99083 | 0.99648 |
| 3086.064 | 0.99107 | 0.99664 |
| 3084.135 | 0.99115 | 0.99679 |
| 3082.206 | 0.9911 | 0.99691 |
| 3080.277 | 0.99101 | 0.99701 |
| 3078.349 | 0.99098 | 0.99709 |
| 3076.42 | 0.99111 | 0.99715 |
| 3074.491 | 0.99143 | 0.99719 |
| 3072.562 | 0.99193 | 0.99722 |
| 3070.633 | 0.99253 | 0.99726 |
| 3068.705 | 0.99312 | 0.99733 |
| 3066.776 | 0.99359 | 0.99739 |
| 3064.847 | 0.99386 | 0.99745 |
| 3062.918 | 0.99392 | 0.99752 |
| 3060.989 | 0.99386 | 0.99762 |
| 3059.061 | 0.99375 | 0.99773 |
| 3057.132 | 0.99372 | 0.99784 |
| 3055.203 | 0.99382 | 0.99797 |
| 3053.274 | 0.9941 | 0.99813 |
| 3051.345 | 0.99449 | 0.99833 |
| 3049.417 | 0.99492 | 0.99854 |
| 3047.488 | 0.99527 | 0.99875 |
| 3045.559 | 0.99547 | 0.99896 |
| 3043.63 | 0.9955 | 0.99918 |
| 3041.702 | 0.99539 | 0.9994 |
| 3039.773 | 0.99523 | 0.99959 |
| 3037.844 | 0.99512 | 0.99971 |
| 3035.915 | 0.99516 | 0.99977 |
| 3033.986 | 0.99536 | 0.99978 |
| 3032.058 | 0.9957 | 0.99976 |
| 3030.129 | 0.9961 | 0.99972 |
| 3028.2 | 0.99646 | 0.99966 |
| 3026.271 | 0.99668 | 0.99961 |
| 3024.342 | 0.99672 | 0.99957 |
| 3022.414 | 0.99662 | 0.99957 |
| 3020.485 | 0.99642 | 0.9996 |
| 3018.556 | 0.99622 | 0.99958 |
| 3016.627 | 0.9961 | 0.99951 |
| 3014.698 | 0.99612 | 0.9994 |
| 3012.77 | 0.99626 | 0.99928 |
| 3010.841 | 0.99646 | 0.9992 |
| 3008.912 | 0.99664 | 0.99913 |
| 3006.983 | 0.99674 | 0.9991 |
| 3005.055 | 0.99671 | 0.99911 |
| 3003.126 | 0.99655 | 0.99918 |
| 3001.197 | 0.99632 | 0.99927 |
| 2999.268 | 0.99611 | 0.99937 |
| 2997.339 | 0.99598 | 0.9994 |
| 2995.411 | 0.99598 | 0.99932 |
| 2993.482 | 0.99611 | 0.99918 |
| 2991.553 | 0.99628 | 0.99899 |
| 2989.624 | 0.99638 | 0.99877 |
| 2987.695 | 0.9963 | 0.99849 |
| 2985.767 | 0.99598 | 0.99813 |
| 2983.838 | 0.99541 | 0.99768 |
| 2981.909 | 0.99466 | 0.99713 |
| 2979.98 | 0.99379 | 0.99652 |
| 2978.051 | 0.99292 | 0.99582 |
| 2976.123 | 0.99212 | 0.99502 |
| 2974.194 | 0.9914 | 0.99411 |
| 2972.265 | 0.99075 | 0.99314 |
| 2970.336 | 0.9901 | 0.99217 |
| 2968.408 | 0.98938 | 0.99124 |
| 2966.479 | 0.98857 | 0.99036 |
| 2964.55 | 0.98771 | 0.98958 |
| 2962.621 | 0.9869 | 0.98896 |
| 2960.692 | 0.98626 | 0.98857 |
| 2958.764 | 0.98586 | 0.98841 |
| 2956.835 | 0.98575 | 0.98845 |
| 2954.906 | 0.98587 | 0.98865 |
| 2952.977 | 0.98608 | 0.9889 |
| 2951.048 | 0.98623 | 0.98916 |
| 2949.12 | 0.98615 | 0.98933 |
| 2947.191 | 0.98574 | 0.98931 |
| 2945.262 | 0.98494 | 0.98899 |
| 2943.333 | 0.98378 | 0.98835 |
| 2941.404 | 0.98235 | 0.98742 |
| 2939.476 | 0.98074 | 0.98622 |
| 2937.547 | 0.97905 | 0.98481 |
| 2935.618 | 0.97733 | 0.98322 |
| 2933.689 | 0.97562 | 0.98156 |
| 2931.76 | 0.97397 | 0.97996 |
| 2929.832 | 0.97242 | 0.97858 |
| 2927.903 | 0.97106 | 0.97757 |
| 2925.974 | 0.97004 | 0.97703 |
| 2924.045 | 0.96955 | 0.97704 |
| 2922.117 | 0.96971 | 0.97759 |
| 2920.188 | 0.9706 | 0.97866 |
| 2918.259 | 0.97219 | 0.98014 |
| 2916.33 | 0.97432 | 0.98186 |
| 2914.401 | 0.97677 | 0.98363 |
| 2912.473 | 0.97926 | 0.98532 |
| 2910.544 | 0.98156 | 0.98685 |
| 2908.615 | 0.98347 | 0.98817 |
| 2906.686 | 0.9849 | 0.98926 |
| 2904.757 | 0.98588 | 0.99016 |
| 2902.829 | 0.98653 | 0.99091 |
| 2900.9 | 0.98702 | 0.99157 |
| 2898.971 | 0.98752 | 0.99216 |
| 2897.042 | 0.98812 | 0.99269 |
| 2895.113 | 0.98886 | 0.99311 |
| 2893.185 | 0.98967 | 0.99343 |
| 2891.256 | 0.99046 | 0.99367 |
| 2889.327 | 0.99111 | 0.99384 |
| 2887.398 | 0.99153 | 0.99394 |
| 2885.47 | 0.99169 | 0.99393 |
| 2883.541 | 0.99162 | 0.99382 |
| 2881.612 | 0.99142 | 0.99363 |
| 2879.683 | 0.99117 | 0.9934 |
| 2877.754 | 0.99098 | 0.99315 |
| 2875.826 | 0.99091 | 0.99289 |
| 2873.897 | 0.99094 | 0.99266 |
| 2871.968 | 0.99096 | 0.99247 |
| 2870.039 | 0.99086 | 0.99233 |
| 2868.11 | 0.9905 | 0.99218 |
| 2866.182 | 0.9898 | 0.99195 |
| 2864.253 | 0.98877 | 0.99156 |
| 2862.324 | 0.98751 | 0.99102 |
| 2860.395 | 0.98623 | 0.9904 |
| 2858.466 | 0.98513 | 0.98981 |
| 2856.538 | 0.98442 | 0.98941 |
| 2854.609 | 0.98426 | 0.98932 |
| 2852.68 | 0.98467 | 0.98961 |
| 2850.751 | 0.9856 | 0.99033 |
| 2848.823 | 0.98691 | 0.99142 |
| 2846.894 | 0.98843 | 0.99274 |
| 2844.965 | 0.99003 | 0.9941 |
| 2843.036 | 0.99161 | 0.99536 |
| 2841.107 | 0.99309 | 0.9964 |
| 2839.179 | 0.99447 | 0.99719 |
| 2837.25 | 0.99571 | 0.9977 |
| 2835.321 | 0.99677 | 0.99799 |
| 2833.392 | 0.99763 | 0.99815 |
| 2831.463 | 0.99823 | 0.99823 |
| 2829.535 | 0.99854 | 0.99832 |
| 2827.606 | 0.99858 | 0.99843 |
| 2825.677 | 0.99844 | 0.99854 |
| 2823.748 | 0.99822 | 0.99863 |
| 2821.819 | 0.99804 | 0.99873 |
| 2819.891 | 0.99802 | 0.99888 |
| 2817.962 | 0.99819 | 0.99908 |
| 2816.033 | 0.99852 | 0.99929 |
| 2814.104 | 0.99893 | 0.99947 |
| 2812.176 | 0.9993 | 0.99961 |
| 2810.247 | 0.99951 | 0.99974 |
| 2808.318 | 0.99952 | 0.99974 |
| 2806.389 | 0.99933 | 0.99973 |
| 2804.46 | 0.99902 | 0.99973 |
| 2802.532 | 0.99872 | 0.99973 |
| 2800.603 | 0.99871 | 0.99973 |
| 2798.674 | 0.99871 | 0.99973 |
| 2796.745 | 0.99871 | 0.99973 |
| 2794.816 | 0.99871 | 0.99972 |
| 2792.888 | 0.99871 | 0.99972 |
| 2790.959 | 0.99871 | 0.99972 |
| 2789.03 | 0.99871 | 0.99972 |
| 2787.101 | 0.99871 | 0.99972 |
| 2785.172 | 0.99871 | 0.99972 |
| 2783.244 | 0.99871 | 0.99971 |
| 2781.315 | 0.99871 | 0.99971 |
| 2779.386 | 0.99871 | 0.99971 |
| 2777.457 | 0.99871 | 0.99971 |
| 2775.529 | 0.99871 | 0.99971 |
| 2773.6 | 0.99871 | 0.9997 |
| 2771.671 | 0.99871 | 0.9997 |
| 2769.742 | 0.99871 | 0.9997 |
| 2767.813 | 0.99871 | 0.9997 |
| 2765.885 | 0.99871 | 0.9997 |
| 2763.956 | 0.99871 | 0.9997 |
| 2762.027 | 0.99871 | 0.99969 |
| 2760.098 | 0.99871 | 0.99969 |
| 2758.169 | 0.99871 | 0.99969 |
| 2756.241 | 0.99871 | 0.99969 |
| 2754.312 | 0.99871 | 0.99969 |
| 2752.383 | 0.99871 | 0.99969 |
| 2750.454 | 0.9987 | 0.99968 |
| 2748.525 | 0.9987 | 0.99968 |
| 2746.597 | 0.9987 | 0.99968 |
| 2744.668 | 0.9987 | 0.99968 |
| 2742.739 | 0.9987 | 0.99968 |
| 2740.81 | 0.9987 | 0.99967 |
| 2738.882 | 0.9987 | 0.99967 |
| 2736.953 | 0.9987 | 0.99967 |
| 2735.024 | 0.9987 | 0.99967 |
| 2733.095 | 0.9987 | 0.99967 |
| 2731.166 | 0.9987 | 0.99967 |
| 2729.238 | 0.9987 | 0.99966 |
| 2727.309 | 0.9987 | 0.99966 |
| 2725.38 | 0.9987 | 0.99966 |
| 2723.451 | 0.9987 | 0.99966 |
| 2721.522 | 0.9987 | 0.99966 |
| 2719.594 | 0.9987 | 0.99966 |
| 2717.665 | 0.9987 | 0.99965 |
| 2715.736 | 0.9987 | 0.99965 |
| 2713.807 | 0.9987 | 0.99965 |
| 2711.878 | 0.9987 | 0.99965 |
| 2709.95 | 0.9987 | 0.99965 |
| 2708.021 | 0.9987 | 0.99964 |
| 2706.092 | 0.9987 | 0.99964 |
| 2704.163 | 0.9987 | 0.99964 |
| 2702.235 | 0.9987 | 0.99964 |
| 2700.306 | 0.99869 | 0.99964 |
| 2698.377 | 0.99869 | 0.99964 |
| 2696.448 | 0.99869 | 0.99963 |
| 2694.519 | 0.99869 | 0.99963 |
| 2692.591 | 0.99869 | 0.99963 |
| 2690.662 | 0.99869 | 0.99963 |
| 2688.733 | 0.99869 | 0.99963 |
| 2686.804 | 0.99869 | 0.99962 |
| 2684.875 | 0.99869 | 0.99962 |
| 2682.947 | 0.99869 | 0.99962 |
| 2681.018 | 0.99869 | 0.99962 |
| 2679.089 | 0.99869 | 0.99962 |
| 2677.16 | 0.99869 | 0.99962 |
| 2675.231 | 0.99869 | 0.99961 |
| 2673.303 | 0.99869 | 0.99961 |
| 2671.374 | 0.99869 | 0.99961 |
| 2669.445 | 0.99869 | 0.99961 |
| 2667.516 | 0.99869 | 0.99961 |
| 2665.588 | 0.99869 | 0.99961 |
| 2663.659 | 0.99869 | 0.9996 |
| 2661.73 | 0.99869 | 0.9996 |
| 2659.801 | 0.99869 | 0.9996 |
| 2657.872 | 0.99869 | 0.9996 |
| 2655.944 | 0.99869 | 0.9996 |
| 2654.015 | 0.99869 | 0.99959 |
| 2652.086 | 0.99868 | 0.99959 |
| 2650.157 | 0.99868 | 0.99959 |
| 2648.228 | 0.99868 | 0.99959 |
| 2646.3 | 0.99868 | 0.99959 |
| 2644.371 | 0.99868 | 0.99959 |
| 2642.442 | 0.99868 | 0.99958 |
| 2640.513 | 0.99868 | 0.99958 |
| 2638.584 | 0.99868 | 0.99958 |
| 2636.656 | 0.99868 | 0.99958 |
| 2634.727 | 0.99868 | 0.99958 |
| 2632.798 | 0.99868 | 0.99958 |
| 2630.869 | 0.99868 | 0.99957 |
| 2628.94 | 0.99868 | 0.99957 |
| 2627.012 | 0.99868 | 0.99957 |
| 2625.083 | 0.99868 | 0.99957 |
| 2623.154 | 0.99868 | 0.99957 |
| 2621.225 | 0.99868 | 0.99956 |
| 2619.297 | 0.99868 | 0.99956 |
| 2617.368 | 0.99868 | 0.99956 |
| 2615.439 | 0.99868 | 0.99956 |
| 2613.51 | 0.99868 | 0.99956 |
| 2611.581 | 0.99868 | 0.99956 |
| 2609.653 | 0.99868 | 0.99955 |
| 2607.724 | 0.99868 | 0.99955 |
| 2605.795 | 0.99868 | 0.99955 |
| 2603.866 | 0.99868 | 0.99955 |
| 2601.937 | 0.99867 | 0.99955 |
| 2600.009 | 0.99867 | 0.99955 |
| 2598.08 | 0.99867 | 0.99954 |
| 2596.151 | 0.99867 | 0.99954 |
| 2594.222 | 0.99867 | 0.99954 |
| 2592.293 | 0.99867 | 0.99954 |
| 2590.365 | 0.99867 | 0.99954 |
| 2588.436 | 0.99867 | 0.99953 |
| 2586.507 | 0.99867 | 0.99953 |
| 2584.578 | 0.99867 | 0.99953 |
| 2582.65 | 0.99867 | 0.99953 |
| 2580.721 | 0.99867 | 0.99953 |
| 2578.792 | 0.99867 | 0.99953 |
| 2576.863 | 0.99867 | 0.99952 |
| 2574.934 | 0.99867 | 0.99952 |
| 2573.006 | 0.99867 | 0.99952 |
| 2571.077 | 0.99867 | 0.99952 |
| 2569.148 | 0.99867 | 0.99952 |
| 2567.219 | 0.99867 | 0.99951 |
| 2565.29 | 0.99867 | 0.99951 |
| 2563.362 | 0.99867 | 0.99951 |
| 2561.433 | 0.99867 | 0.99951 |
| 2559.504 | 0.99867 | 0.99951 |
| 2557.575 | 0.99867 | 0.99951 |
| 2555.646 | 0.99867 | 0.9995 |
| 2553.718 | 0.99867 | 0.9995 |
| 2551.789 | 0.99866 | 0.9995 |
| 2549.86 | 0.99866 | 0.9995 |
| 2547.931 | 0.99866 | 0.9995 |
| 2546.003 | 0.99866 | 0.9995 |
| 2544.074 | 0.99866 | 0.99949 |
| 2542.145 | 0.99866 | 0.99949 |
| 2540.216 | 0.99866 | 0.99949 |
| 2538.287 | 0.99866 | 0.99949 |
| 2536.359 | 0.99866 | 0.99949 |
| 2534.43 | 0.99866 | 0.99948 |
| 2532.501 | 0.99866 | 0.99948 |
| 2530.572 | 0.99866 | 0.99948 |
| 2528.643 | 0.99866 | 0.99948 |
| 2526.715 | 0.99866 | 0.99948 |
| 2524.786 | 0.99866 | 0.99948 |
| 2522.857 | 0.99866 | 0.99947 |
| 2520.928 | 0.99866 | 0.99947 |
| 2518.999 | 0.99866 | 0.99947 |
| 2517.071 | 0.99866 | 0.99947 |
| 2515.142 | 0.99866 | 0.99947 |
| 2513.213 | 0.99866 | 0.99947 |
| 2511.284 | 0.99866 | 0.99946 |
| 2509.356 | 0.99866 | 0.99946 |
| 2507.427 | 0.99866 | 0.99946 |
| 2505.498 | 0.99866 | 0.99946 |
| 2503.569 | 0.99865 | 0.99946 |
| 2501.64 | 0.99865 | 0.99945 |
| 2499.712 | 0.99865 | 0.99945 |
| 2497.783 | 0.99865 | 0.99945 |
| 2495.854 | 0.99865 | 0.99945 |
| 2493.925 | 0.99865 | 0.99945 |
| 2491.996 | 0.99865 | 0.99945 |
| 2490.068 | 0.99865 | 0.99944 |
| 2488.139 | 0.99865 | 0.99944 |
| 2486.21 | 0.99865 | 0.99944 |
| 2484.281 | 0.99865 | 0.99944 |
| 2482.352 | 0.99865 | 0.99944 |
| 2480.424 | 0.99865 | 0.99944 |
| 2478.495 | 0.99865 | 0.99943 |
| 2476.566 | 0.99865 | 0.99943 |
| 2474.637 | 0.99865 | 0.99943 |
| 2472.709 | 0.99865 | 0.99943 |
| 2470.78 | 0.99865 | 0.99943 |
| 2468.851 | 0.99865 | 0.99942 |
| 2466.922 | 0.99865 | 0.99942 |
| 2464.993 | 0.99865 | 0.99942 |
| 2463.065 | 0.99865 | 0.99942 |
| 2461.136 | 0.99865 | 0.99942 |
| 2459.207 | 0.99865 | 0.99942 |
| 2457.278 | 0.99865 | 0.99941 |
| 2455.349 | 0.99865 | 0.99941 |
| 2453.421 | 0.99864 | 0.99941 |
| 2451.492 | 0.99864 | 0.99941 |
| 2449.563 | 0.99864 | 0.99941 |
| 2447.634 | 0.99864 | 0.9994 |
| 2445.705 | 0.99864 | 0.9994 |
| 2443.777 | 0.99864 | 0.9994 |
| 2441.848 | 0.99864 | 0.9994 |
| 2439.919 | 0.99864 | 0.9994 |
| 2437.99 | 0.99864 | 0.9994 |
| 2436.062 | 0.99864 | 0.99939 |
| 2434.133 | 0.99864 | 0.99939 |
| 2432.204 | 0.99864 | 0.99939 |
| 2430.275 | 0.99864 | 0.99939 |
| 2428.346 | 0.99864 | 0.99939 |
| 2426.418 | 0.99864 | 0.99939 |
| 2424.489 | 0.99864 | 0.99938 |
| 2422.56 | 0.99864 | 0.99938 |
| 2420.631 | 0.99864 | 0.99938 |
| 2418.702 | 0.99864 | 0.99938 |
| 2416.774 | 0.99864 | 0.99938 |
| 2414.845 | 0.99864 | 0.99937 |
| 2412.916 | 0.99864 | 0.99937 |
| 2410.987 | 0.99864 | 0.99937 |
| 2409.058 | 0.99864 | 0.99937 |
| 2407.13 | 0.99864 | 0.99937 |
| 2405.201 | 0.99863 | 0.99937 |
| 2403.272 | 0.99863 | 0.99936 |
| 2401.343 | 0.99863 | 0.99936 |
| 2399.415 | 0.99863 | 0.99936 |
| 2397.486 | 0.99863 | 0.99936 |
| 2395.557 | 0.99863 | 0.99936 |
| 2393.628 | 0.99863 | 0.99936 |
| 2391.699 | 0.99863 | 0.99935 |
| 2389.771 | 0.99863 | 0.99935 |
| 2387.842 | 0.99863 | 0.99935 |
| 2385.913 | 0.99863 | 0.99935 |
| 2383.984 | 0.99863 | 0.99935 |
| 2382.055 | 0.99863 | 0.99934 |
| 2380.127 | 0.99863 | 0.99934 |
| 2378.198 | 0.99863 | 0.99934 |
| 2376.269 | 0.99863 | 0.99934 |
| 2374.34 | 0.99863 | 0.99934 |
| 2372.411 | 0.99863 | 0.99934 |
| 2370.483 | 0.99863 | 0.99933 |
| 2368.554 | 0.99863 | 0.99933 |
| 2366.625 | 0.99863 | 0.99933 |
| 2364.696 | 0.99863 | 0.99933 |
| 2362.768 | 0.99863 | 0.99933 |
| 2360.839 | 0.99863 | 0.99933 |
| 2358.91 | 0.99863 | 0.99932 |
| 2356.981 | 0.99863 | 0.99932 |
| 2355.052 | 0.99862 | 0.99932 |
| 2353.124 | 0.99862 | 0.99932 |
| 2351.195 | 0.99862 | 0.99932 |
| 2349.266 | 0.99862 | 0.99931 |
| 2347.337 | 0.99862 | 0.99931 |
| 2345.408 | 0.99862 | 0.99931 |
| 2343.48 | 0.99862 | 0.99931 |
| 2341.551 | 0.99862 | 0.99931 |
| 2339.622 | 0.99862 | 0.99931 |
| 2337.693 | 0.99862 | 0.9993 |
| 2335.764 | 0.99862 | 0.9993 |
| 2333.836 | 0.99862 | 0.9993 |
| 2331.907 | 0.99862 | 0.9993 |
| 2329.978 | 0.99862 | 0.9993 |
| 2328.049 | 0.99862 | 0.99929 |
| 2326.12 | 0.99862 | 0.99929 |
| 2324.192 | 0.99862 | 0.99929 |
| 2322.263 | 0.99862 | 0.99929 |
| 2320.334 | 0.99862 | 0.99929 |
| 2318.405 | 0.99862 | 0.99929 |
| 2316.477 | 0.99862 | 0.99928 |
| 2314.548 | 0.99862 | 0.99928 |
| 2312.619 | 0.99862 | 0.99928 |
| 2310.69 | 0.99862 | 0.99928 |
| 2308.761 | 0.99862 | 0.99928 |
| 2306.833 | 0.99862 | 0.99928 |
| 2304.904 | 0.99861 | 0.99927 |
| 2302.975 | 0.99861 | 0.99927 |
| 2301.046 | 0.99861 | 0.99927 |
| 2299.117 | 0.99861 | 0.99927 |
| 2297.189 | 0.99861 | 0.99927 |
| 2295.26 | 0.99861 | 0.99926 |
| 2293.331 | 0.99861 | 0.99926 |
| 2291.402 | 0.99861 | 0.99926 |
| 2289.473 | 0.99861 | 0.99926 |
| 2287.545 | 0.99861 | 0.99926 |
| 2285.616 | 0.99861 | 0.99926 |
| 2283.687 | 0.99861 | 0.99925 |
| 2281.758 | 0.99861 | 0.99925 |
| 2279.83 | 0.99861 | 0.99925 |
| 2277.901 | 0.99861 | 0.99925 |
| 2275.972 | 0.99861 | 0.99925 |
| 2274.043 | 0.99861 | 0.99925 |
| 2272.114 | 0.99861 | 0.99924 |
| 2270.186 | 0.99861 | 0.99924 |
| 2268.257 | 0.99861 | 0.99924 |
| 2266.328 | 0.99861 | 0.99924 |
| 2264.399 | 0.99861 | 0.99924 |
| 2262.47 | 0.99861 | 0.99923 |
| 2260.542 | 0.99861 | 0.99923 |
| 2258.613 | 0.99861 | 0.99923 |
| 2256.684 | 0.9986 | 0.99923 |
| 2254.755 | 0.9986 | 0.99923 |
| 2252.826 | 0.9986 | 0.99923 |
| 2250.898 | 0.9986 | 0.99922 |
| 2248.969 | 0.9986 | 0.99922 |
| 2247.04 | 0.9986 | 0.99922 |
| 2245.111 | 0.9986 | 0.99922 |
| 2243.183 | 0.9986 | 0.99922 |
| 2241.254 | 0.9986 | 0.99922 |
| 2239.325 | 0.9986 | 0.99921 |
| 2237.396 | 0.9986 | 0.99921 |
| 2235.467 | 0.9986 | 0.99921 |
| 2233.539 | 0.9986 | 0.99921 |
| 2231.61 | 0.9986 | 0.99921 |
| 2229.681 | 0.9986 | 0.9992 |
| 2227.752 | 0.9986 | 0.9992 |
| 2225.823 | 0.9986 | 0.9992 |
| 2223.895 | 0.9986 | 0.9992 |
| 2221.966 | 0.9986 | 0.9992 |
| 2220.037 | 0.9986 | 0.9992 |
| 2218.108 | 0.9986 | 0.99919 |
| 2216.179 | 0.9986 | 0.99919 |
| 2214.251 | 0.9986 | 0.99919 |
| 2212.322 | 0.9986 | 0.99919 |
| 2210.393 | 0.9986 | 0.99919 |
| 2208.464 | 0.9986 | 0.99919 |
| 2206.536 | 0.99859 | 0.99918 |
| 2204.607 | 0.99859 | 0.99918 |
| 2202.678 | 0.99859 | 0.99918 |
| 2200.749 | 0.99859 | 0.99918 |
| 2198.82 | 0.99859 | 0.99918 |
| 2196.892 | 0.99859 | 0.99917 |
| 2194.963 | 0.99859 | 0.99917 |
| 2193.034 | 0.99859 | 0.99917 |
| 2191.105 | 0.99859 | 0.99917 |
| 2189.176 | 0.99859 | 0.99917 |
| 2187.248 | 0.99859 | 0.99917 |
| 2185.319 | 0.99859 | 0.99916 |
| 2183.39 | 0.99859 | 0.99916 |
| 2181.461 | 0.99859 | 0.99916 |
| 2179.532 | 0.99859 | 0.99916 |
| 2177.604 | 0.99859 | 0.99916 |
| 2175.675 | 0.99859 | 0.99915 |
| 2173.746 | 0.99859 | 0.99915 |
| 2171.817 | 0.99859 | 0.99915 |
| 2169.889 | 0.99859 | 0.99915 |
| 2167.96 | 0.99859 | 0.99915 |
| 2166.031 | 0.99859 | 0.99915 |
| 2164.102 | 0.99859 | 0.99914 |
| 2162.173 | 0.99859 | 0.99914 |
| 2160.245 | 0.99859 | 0.99914 |
| 2158.316 | 0.99859 | 0.99914 |
| 2156.387 | 0.99858 | 0.99914 |
| 2154.458 | 0.99858 | 0.99914 |
| 2152.529 | 0.99858 | 0.99913 |
| 2150.601 | 0.99858 | 0.99913 |
| 2148.672 | 0.99858 | 0.99913 |
| 2146.743 | 0.99858 | 0.99913 |
| 2144.814 | 0.99858 | 0.99913 |
| 2142.885 | 0.99858 | 0.99912 |
| 2140.957 | 0.99858 | 0.99912 |
| 2139.028 | 0.99858 | 0.99912 |
| 2137.099 | 0.99858 | 0.99912 |
| 2135.17 | 0.99858 | 0.99912 |
| 2133.242 | 0.99858 | 0.99912 |
| 2131.313 | 0.99858 | 0.99911 |
| 2129.384 | 0.99858 | 0.99911 |
| 2127.455 | 0.99858 | 0.99911 |
| 2125.526 | 0.99858 | 0.99911 |
| 2123.598 | 0.99858 | 0.99911 |
| 2121.669 | 0.99858 | 0.99911 |
| 2119.74 | 0.99858 | 0.9991 |
| 2117.811 | 0.99858 | 0.9991 |
| 2115.882 | 0.99858 | 0.9991 |
| 2113.954 | 0.99858 | 0.9991 |
| 2112.025 | 0.99858 | 0.9991 |
| 2110.096 | 0.99858 | 0.99909 |
| 2108.167 | 0.99857 | 0.99909 |
| 2106.238 | 0.99857 | 0.99909 |
| 2104.31 | 0.99857 | 0.99909 |
| 2102.381 | 0.99857 | 0.99909 |
| 2100.452 | 0.99857 | 0.99909 |
| 2098.523 | 0.99857 | 0.99908 |
| 2096.595 | 0.99857 | 0.99908 |
| 2094.666 | 0.99857 | 0.99908 |
| 2092.737 | 0.99857 | 0.99908 |
| 2090.808 | 0.99857 | 0.99908 |
| 2088.879 | 0.99857 | 0.99908 |
| 2086.951 | 0.99857 | 0.99907 |
| 2085.022 | 0.99857 | 0.99907 |
| 2083.093 | 0.99857 | 0.99907 |
| 2081.164 | 0.99857 | 0.99907 |
| 2079.235 | 0.99857 | 0.99907 |
| 2077.307 | 0.99857 | 0.99906 |
| 2075.378 | 0.99857 | 0.99906 |
| 2073.449 | 0.99857 | 0.99906 |
| 2071.52 | 0.99857 | 0.99906 |
| 2069.591 | 0.99857 | 0.99906 |
| 2067.663 | 0.99857 | 0.99906 |
| 2065.734 | 0.99857 | 0.99905 |
| 2063.805 | 0.99857 | 0.99905 |
| 2061.876 | 0.99857 | 0.99905 |
| 2059.948 | 0.99857 | 0.99905 |
| 2058.019 | 0.99856 | 0.99905 |
| 2056.09 | 0.99856 | 0.99904 |
| 2054.161 | 0.99856 | 0.99904 |
| 2052.232 | 0.99856 | 0.99904 |
| 2050.304 | 0.99856 | 0.99904 |
| 2048.375 | 0.99856 | 0.99904 |
| 2046.446 | 0.99856 | 0.99904 |
| 2044.517 | 0.99856 | 0.99903 |
| 2042.588 | 0.99856 | 0.99903 |
| 2040.66 | 0.99856 | 0.99903 |
| 2038.731 | 0.99856 | 0.99903 |
| 2036.802 | 0.99856 | 0.99903 |
| 2034.873 | 0.99856 | 0.99903 |
| 2032.944 | 0.99856 | 0.99902 |
| 2031.016 | 0.99856 | 0.99902 |
| 2029.087 | 0.99856 | 0.99902 |
| 2027.158 | 0.99856 | 0.99902 |
| 2025.229 | 0.99856 | 0.99902 |
| 2023.301 | 0.99856 | 0.99901 |
| 2021.372 | 0.99856 | 0.99901 |
| 2019.443 | 0.99856 | 0.99901 |
| 2017.514 | 0.99856 | 0.99901 |
| 2015.585 | 0.99856 | 0.99901 |
| 2013.657 | 0.99856 | 0.99901 |
| 2011.728 | 0.99856 | 0.999 |
| 2009.799 | 0.99856 | 0.999 |
| 2007.87 | 0.99855 | 0.999 |
| 2005.941 | 0.99855 | 0.999 |
| 2004.013 | 0.99855 | 0.999 |
| 2002.084 | 0.99855 | 0.999 |
| 2000.155 | 0.99855 | 0.99899 |
| 1998.226 | 0.99855 | 0.99899 |
| 1996.297 | 0.99855 | 0.99899 |
| 1994.369 | 0.99855 | 0.99899 |
| 1992.44 | 0.99855 | 0.99899 |
| 1990.511 | 0.99855 | 0.99898 |
| 1988.582 | 0.99855 | 0.99898 |
| 1986.653 | 0.99855 | 0.99898 |
| 1984.725 | 0.99855 | 0.99898 |
| 1982.796 | 0.99855 | 0.99898 |
| 1980.867 | 0.99855 | 0.99898 |
| 1978.938 | 0.99855 | 0.99897 |
| 1977.01 | 0.99855 | 0.99897 |
| 1975.081 | 0.99855 | 0.99897 |
| 1973.152 | 0.99855 | 0.99897 |
| 1971.223 | 0.99855 | 0.99897 |
| 1969.294 | 0.99855 | 0.99897 |
| 1967.366 | 0.99855 | 0.99896 |
| 1965.437 | 0.99855 | 0.99896 |
| 1963.508 | 0.99855 | 0.99896 |
| 1961.579 | 0.99855 | 0.99896 |
| 1959.65 | 0.99854 | 0.99896 |
| 1957.722 | 0.99854 | 0.99895 |
| 1955.793 | 0.99854 | 0.99895 |
| 1953.864 | 0.99854 | 0.99895 |
| 1951.935 | 0.99854 | 0.99895 |
| 1950.006 | 0.99854 | 0.99895 |
| 1948.078 | 0.99854 | 0.99895 |
| 1946.149 | 0.99854 | 0.99894 |
| 1944.22 | 0.99854 | 0.99894 |
| 1942.291 | 0.99854 | 0.99894 |
| 1940.363 | 0.99854 | 0.99894 |
| 1938.434 | 0.99854 | 0.99894 |
| 1936.505 | 0.99854 | 0.99893 |
| 1934.576 | 0.99854 | 0.99893 |
| 1932.647 | 0.99854 | 0.99893 |
| 1930.719 | 0.99854 | 0.99893 |
| 1928.79 | 0.99854 | 0.99893 |
| 1926.861 | 0.99854 | 0.99893 |
| 1924.932 | 0.99854 | 0.99892 |
| 1923.003 | 0.99863 | 0.99892 |
| 1921.075 | 0.99858 | 0.99892 |
| 1919.146 | 0.99841 | 0.99892 |
| 1917.217 | 0.9982 | 0.99892 |
| 1915.288 | 0.998 | 0.99892 |
| 1913.359 | 0.99784 | 0.99891 |
| 1911.431 | 0.99778 | 0.99891 |
| 1909.502 | 0.99786 | 0.99891 |
| 1907.573 | 0.99798 | 0.99891 |
| 1905.644 | 0.99804 | 0.99891 |
| 1903.716 | 0.99801 | 0.9989 |
| 1901.787 | 0.99788 | 0.9989 |
| 1899.858 | 0.99765 | 0.9989 |
| 1897.929 | 0.99737 | 0.9989 |
| 1896 | 0.99717 | 0.9989 |
| 1894.072 | 0.99711 | 0.9989 |
| 1892.143 | 0.99717 | 0.99889 |
| 1890.214 | 0.99734 | 0.99889 |
| 1888.285 | 0.99752 | 0.99889 |
| 1886.356 | 0.99758 | 0.99889 |
| 1884.428 | 0.99749 | 0.99889 |
| 1882.499 | 0.99732 | 0.99889 |
| 1880.57 | 0.99707 | 0.99888 |
| 1878.641 | 0.9968 | 0.99888 |
| 1876.712 | 0.9966 | 0.99888 |
| 1874.784 | 0.99654 | 0.99888 |
| 1872.855 | 0.99662 | 0.99888 |
| 1870.926 | 0.99679 | 0.99887 |
| 1868.997 | 0.99707 | 0.99887 |
| 1867.069 | 0.99736 | 0.99887 |
| 1865.14 | 0.99747 | 0.99887 |
| 1863.211 | 0.99726 | 0.99887 |
| 1861.282 | 0.9968 | 0.99887 |
| 1859.353 | 0.99629 | 0.99886 |
| 1857.425 | 0.99584 | 0.99886 |
| 1855.496 | 0.99555 | 0.99886 |
| 1853.567 | 0.99552 | 0.99886 |
| 1851.638 | 0.99576 | 0.99886 |
| 1849.709 | 0.99609 | 0.99886 |
| 1847.781 | 0.99636 | 0.99885 |
| 1845.852 | 0.99659 | 0.99885 |
| 1843.923 | 0.99674 | 0.99885 |
| 1841.994 | 0.9967 | 0.99885 |
| 1840.065 | 0.99647 | 0.99885 |
| 1838.137 | 0.99624 | 0.99884 |
| 1836.208 | 0.99619 | 0.99884 |
| 1834.279 | 0.99632 | 0.99884 |
| 1832.35 | 0.99662 | 0.99884 |
| 1830.422 | 0.99707 | 0.99884 |
| 1828.493 | 0.99752 | 0.99884 |
| 1826.564 | 0.99776 | 0.99883 |
| 1824.635 | 0.99776 | 0.99883 |
| 1822.706 | 0.99762 | 0.99883 |
| 1820.778 | 0.99736 | 0.99883 |
| 1818.849 | 0.997 | 0.99883 |
| 1816.92 | 0.99671 | 0.99883 |
| 1814.991 | 0.99662 | 0.99882 |
| 1813.062 | 0.99672 | 0.99882 |
| 1811.134 | 0.99689 | 0.99882 |
| 1809.205 | 0.99711 | 0.99882 |
| 1807.276 | 0.99739 | 0.99882 |
| 1805.347 | 0.99758 | 0.99881 |
| 1803.418 | 0.99763 | 0.99882 |
| 1801.49 | 0.99755 | 0.99875 |
| 1799.561 | 0.99736 | 0.99847 |
| 1797.632 | 0.99703 | 0.99816 |
| 1795.703 | 0.99663 | 0.99783 |
| 1793.775 | 0.99632 | 0.99743 |
| 1791.846 | 0.99614 | 0.99713 |
| 1789.917 | 0.99599 | 0.99694 |
| 1787.988 | 0.99583 | 0.99657 |
| 1786.059 | 0.99572 | 0.99612 |
| 1784.131 | 0.9957 | 0.9961 |
| 1782.202 | 0.99564 | 0.99658 |
| 1780.273 | 0.99549 | 0.99714 |
| 1778.344 | 0.99528 | 0.99729 |
| 1776.415 | 0.99501 | 0.99709 |
| 1774.487 | 0.99468 | 0.99711 |
| 1772.558 | 0.99445 | 0.99731 |
| 1770.629 | 0.99435 | 0.99728 |
| 1768.7 | 0.99424 | 0.99709 |
| 1766.771 | 0.99399 | 0.99665 |
| 1764.843 | 0.99359 | 0.99596 |
| 1762.914 | 0.99311 | 0.9956 |
| 1760.985 | 0.99254 | 0.99565 |
| 1759.056 | 0.99188 | 0.99536 |
| 1757.128 | 0.99121 | 0.99459 |
| 1755.199 | 0.99054 | 0.9936 |
| 1753.27 | 0.98975 | 0.99268 |
| 1751.341 | 0.9889 | 0.99203 |
| 1749.412 | 0.98823 | 0.99118 |
| 1747.484 | 0.9877 | 0.99022 |
| 1745.555 | 0.98717 | 0.98989 |
| 1743.626 | 0.98659 | 0.98969 |
| 1741.697 | 0.98602 | 0.98922 |
| 1739.768 | 0.98552 | 0.98896 |
| 1737.84 | 0.9849 | 0.98887 |
| 1735.911 | 0.9841 | 0.98857 |
| 1733.982 | 0.98333 | 0.98795 |
| 1732.053 | 0.98266 | 0.98718 |
| 1730.124 | 0.982 | 0.98683 |
| 1728.196 | 0.98142 | 0.98681 |
| 1726.267 | 0.98101 | 0.98642 |
| 1724.338 | 0.98069 | 0.98596 |
| 1722.409 | 0.98031 | 0.98582 |
| 1720.481 | 0.97979 | 0.98543 |
| 1718.552 | 0.9792 | 0.98485 |
| 1716.623 | 0.97865 | 0.98437 |
| 1714.694 | 0.97811 | 0.98376 |
| 1712.765 | 0.97769 | 0.9834 |
| 1710.837 | 0.97764 | 0.98322 |
| 1708.908 | 0.97791 | 0.98305 |
| 1706.979 | 0.9783 | 0.98343 |
| 1705.05 | 0.97849 | 0.98373 |
| 1703.121 | 0.97812 | 0.98323 |
| 1701.193 | 0.97722 | 0.98228 |
| 1699.264 | 0.97602 | 0.9812 |
| 1697.335 | 0.97471 | 0.98047 |
| 1695.406 | 0.97353 | 0.98032 |
| 1693.477 | 0.97266 | 0.97979 |
| 1691.549 | 0.97213 | 0.97865 |
| 1689.62 | 0.97178 | 0.97796 |
| 1687.691 | 0.97136 | 0.97789 |
| 1685.762 | 0.97084 | 0.97789 |
| 1683.833 | 0.9703 | 0.97772 |
| 1681.905 | 0.96949 | 0.97742 |
| 1679.976 | 0.96825 | 0.97715 |
| 1678.047 | 0.96698 | 0.9766 |
| 1676.118 | 0.96607 | 0.97588 |
| 1674.19 | 0.96547 | 0.97571 |
| 1672.261 | 0.9648 | 0.97563 |
| 1670.332 | 0.96404 | 0.97435 |
| 1668.403 | 0.96339 | 0.97221 |
| 1666.474 | 0.96258 | 0.97091 |
| 1664.546 | 0.9614 | 0.9711 |
| 1662.617 | 0.96032 | 0.97079 |
| 1660.688 | 0.95967 | 0.96944 |
| 1658.759 | 0.95922 | 0.9689 |
| 1656.83 | 0.95869 | 0.96918 |
| 1654.902 | 0.95822 | 0.96895 |
| 1652.973 | 0.95797 | 0.96838 |
| 1651.044 | 0.95766 | 0.96793 |
| 1649.115 | 0.95704 | 0.96752 |
| 1647.186 | 0.95638 | 0.96729 |
| 1645.258 | 0.9559 | 0.96715 |
| 1643.329 | 0.95538 | 0.96739 |
| 1641.4 | 0.95477 | 0.96796 |
| 1639.471 | 0.95437 | 0.96775 |
| 1637.543 | 0.95433 | 0.96736 |
| 1635.614 | 0.95452 | 0.96803 |
| 1633.685 | 0.95482 | 0.96894 |
| 1631.756 | 0.9554 | 0.96898 |
| 1629.827 | 0.95629 | 0.96897 |
| 1627.899 | 0.95726 | 0.96969 |
| 1625.97 | 0.95817 | 0.97077 |
| 1624.041 | 0.95915 | 0.97173 |
| 1622.112 | 0.9602 | 0.97221 |
| 1620.183 | 0.9612 | 0.97244 |
| 1618.255 | 0.96217 | 0.97289 |
| 1616.326 | 0.96331 | 0.97347 |
| 1614.397 | 0.96469 | 0.97393 |
| 1612.468 | 0.96622 | 0.97434 |
| 1610.539 | 0.9678 | 0.97457 |
| 1608.611 | 0.96932 | 0.97467 |
| 1606.682 | 0.97064 | 0.97521 |
| 1604.753 | 0.97168 | 0.97606 |
| 1602.824 | 0.9725 | 0.97652 |
| 1600.896 | 0.97326 | 0.97684 |
| 1598.967 | 0.97401 | 0.9775 |
| 1597.038 | 0.97475 | 0.97841 |
| 1595.109 | 0.97555 | 0.97931 |
| 1593.18 | 0.97645 | 0.98009 |
| 1591.252 | 0.97743 | 0.98073 |
| 1589.323 | 0.97831 | 0.98162 |
| 1587.394 | 0.97895 | 0.98291 |
| 1585.465 | 0.97936 | 0.98385 |
| 1583.536 | 0.97964 | 0.98433 |
| 1581.608 | 0.97987 | 0.98501 |
| 1579.679 | 0.97998 | 0.98588 |
| 1577.75 | 0.97997 | 0.98649 |
| 1575.821 | 0.98003 | 0.9866 |
| 1573.892 | 0.98027 | 0.98631 |
| 1571.964 | 0.98061 | 0.98641 |
| 1570.035 | 0.98113 | 0.98712 |
| 1568.106 | 0.98181 | 0.98747 |
| 1566.177 | 0.98243 | 0.98756 |
| 1564.249 | 0.98272 | 0.98763 |
| 1562.32 | 0.98256 | 0.98721 |
| 1560.391 | 0.98208 | 0.98663 |
| 1558.462 | 0.9814 | 0.98633 |
| 1556.533 | 0.98067 | 0.98591 |
| 1554.605 | 0.98018 | 0.9852 |
| 1552.676 | 0.98012 | 0.98487 |
| 1550.747 | 0.98047 | 0.98522 |
| 1548.818 | 0.98111 | 0.98584 |
| 1546.889 | 0.98189 | 0.98637 |
| 1544.961 | 0.98251 | 0.98658 |
| 1543.032 | 0.98276 | 0.98667 |
| 1541.103 | 0.98262 | 0.98671 |
| 1539.174 | 0.98218 | 0.98675 |
| 1537.245 | 0.98172 | 0.98667 |
| 1535.317 | 0.98153 | 0.98643 |
| 1533.388 | 0.98186 | 0.98655 |
| 1531.459 | 0.98275 | 0.98711 |
| 1529.53 | 0.98393 | 0.98799 |
| 1527.602 | 0.98508 | 0.98875 |
| 1525.673 | 0.98584 | 0.98872 |
| 1523.744 | 0.98607 | 0.98846 |
| 1521.815 | 0.98603 | 0.98846 |
| 1519.886 | 0.98607 | 0.98873 |
| 1517.958 | 0.98641 | 0.98948 |
| 1516.029 | 0.98709 | 0.99012 |
| 1514.1 | 0.98802 | 0.99008 |
| 1512.171 | 0.98914 | 0.99 |
| 1510.242 | 0.99023 | 0.99059 |
| 1508.314 | 0.99109 | 0.9915 |
| 1506.385 | 0.99172 | 0.99209 |
| 1504.456 | 0.99207 | 0.99231 |
| 1502.527 | 0.99205 | 0.99252 |
| 1500.598 | 0.99198 | 0.99296 |
| 1498.67 | 0.99232 | 0.99367 |
| 1496.741 | 0.99317 | 0.99487 |
| 1494.812 | 0.99428 | 0.99624 |
| 1492.883 | 0.99532 | 0.99682 |
| 1490.955 | 0.99608 | 0.99671 |
| 1489.026 | 0.99651 | 0.99677 |
| 1487.097 | 0.99661 | 0.99728 |
| 1485.168 | 0.99651 | 0.99802 |
| 1483.239 | 0.99643 | 0.9984 |
| 1481.311 | 0.99645 | 0.99814 |
| 1479.382 | 0.99655 | 0.99786 |
| 1477.453 | 0.99654 | 0.99797 |
| 1475.524 | 0.99631 | 0.99795 |
| 1473.595 | 0.99593 | 0.99719 |
| 1471.667 | 0.99537 | 0.99607 |
| 1469.738 | 0.99449 | 0.99552 |
| 1467.809 | 0.99335 | 0.99543 |
| 1465.88 | 0.99227 | 0.99501 |
| 1463.951 | 0.99148 | 0.99441 |
| 1462.023 | 0.99092 | 0.99421 |
| 1460.094 | 0.99058 | 0.99426 |
| 1458.165 | 0.99065 | 0.99407 |
| 1456.236 | 0.991 | 0.99382 |
| 1454.308 | 0.9912 | 0.99381 |
| 1452.379 | 0.99118 | 0.99354 |
| 1450.45 | 0.99116 | 0.99294 |
| 1448.521 | 0.99119 | 0.99298 |
| 1446.592 | 0.99117 | 0.99384 |
| 1444.664 | 0.99116 | 0.99444 |
| 1442.735 | 0.99127 | 0.99434 |
| 1440.806 | 0.99145 | 0.99424 |
| 1438.877 | 0.99156 | 0.99441 |
| 1436.948 | 0.99168 | 0.99467 |
| 1435.02 | 0.99195 | 0.99474 |
| 1433.091 | 0.99227 | 0.99456 |
| 1431.162 | 0.99244 | 0.99454 |
| 1429.233 | 0.99244 | 0.99458 |
| 1427.304 | 0.99226 | 0.99452 |
| 1425.376 | 0.99189 | 0.99452 |
| 1423.447 | 0.99133 | 0.99431 |
| 1421.518 | 0.99065 | 0.99378 |
| 1419.589 | 0.98992 | 0.99329 |
| 1417.661 | 0.98912 | 0.9929 |
| 1415.732 | 0.9882 | 0.99234 |
| 1413.803 | 0.98717 | 0.99151 |
| 1411.874 | 0.98604 | 0.99059 |
| 1409.945 | 0.98482 | 0.98983 |
| 1408.017 | 0.98352 | 0.98939 |
| 1406.088 | 0.98232 | 0.98899 |
| 1404.159 | 0.98169 | 0.98828 |
| 1402.23 | 0.98186 | 0.98757 |
| 1400.301 | 0.98204 | 0.98746 |
| 1398.373 | 0.98043 | 0.98754 |
| 1396.444 | 0.97508 | 0.98583 |
| 1394.515 | 0.96481 | 0.98046 |
| 1392.586 | 0.94981 | 0.97149 |
| 1390.657 | 0.93197 | 0.96107 |
| 1388.729 | 0.91461 | 0.95189 |
| 1386.8 | 0.90121 | 0.94552 |
| 1384.871 | 0.89419 | 0.94251 |
| 1382.942 | 0.89468 | 0.94297 |
| 1381.013 | 0.9028 | 0.9468 |
| 1379.085 | 0.91735 | 0.95393 |
| 1377.156 | 0.93561 | 0.96378 |
| 1375.227 | 0.954 | 0.97443 |
| 1373.298 | 0.96941 | 0.98284 |
| 1371.37 | 0.98006 | 0.98737 |
| 1369.441 | 0.98577 | 0.98902 |
| 1367.512 | 0.98781 | 0.98984 |
| 1365.583 | 0.98821 | 0.99079 |
| 1363.654 | 0.98852 | 0.99181 |
| 1361.726 | 0.98921 | 0.99259 |
| 1359.797 | 0.99006 | 0.99304 |
| 1357.868 | 0.99087 | 0.99332 |
| 1355.939 | 0.99167 | 0.99349 |
| 1354.01 | 0.99248 | 0.99354 |
| 1352.082 | 0.9932 | 0.99375 |
| 1350.153 | 0.99374 | 0.99408 |
| 1348.224 | 0.99404 | 0.99427 |
| 1346.295 | 0.99414 | 0.99442 |
| 1344.366 | 0.9941 | 0.99469 |
| 1342.438 | 0.99404 | 0.99498 |
| 1340.509 | 0.99411 | 0.99517 |
| 1338.58 | 0.99439 | 0.99528 |
| 1336.651 | 0.99477 | 0.99531 |
| 1334.723 | 0.99514 | 0.99525 |
| 1332.794 | 0.99538 | 0.99508 |
| 1330.865 | 0.99539 | 0.99488 |
| 1328.936 | 0.99513 | 0.99474 |
| 1327.007 | 0.99464 | 0.99462 |
| 1325.079 | 0.99403 | 0.99447 |
| 1323.15 | 0.99342 | 0.99426 |
| 1321.221 | 0.99292 | 0.99397 |
| 1319.292 | 0.99261 | 0.99358 |
| 1317.363 | 0.99251 | 0.99311 |
| 1315.435 | 0.99255 | 0.99261 |
| 1313.506 | 0.9926 | 0.99209 |
| 1311.577 | 0.99253 | 0.99154 |
| 1309.648 | 0.99224 | 0.99098 |
| 1307.719 | 0.99173 | 0.99046 |
| 1305.791 | 0.99106 | 0.98999 |
| 1303.862 | 0.99036 | 0.98956 |
| 1301.933 | 0.98973 | 0.98916 |
| 1300.004 | 0.98925 | 0.98877 |
| 1298.076 | 0.98894 | 0.98838 |
| 1296.147 | 0.98875 | 0.98803 |
| 1294.218 | 0.9886 | 0.98773 |
| 1292.289 | 0.98836 | 0.98747 |
| 1290.36 | 0.98798 | 0.98724 |
| 1288.432 | 0.98745 | 0.98701 |
| 1286.503 | 0.98683 | 0.98678 |
| 1284.574 | 0.98622 | 0.98654 |
| 1282.645 | 0.98572 | 0.98624 |
| 1280.716 | 0.98539 | 0.98583 |
| 1278.788 | 0.98523 | 0.9853 |
| 1276.859 | 0.98518 | 0.98475 |
| 1274.93 | 0.98513 | 0.98421 |
| 1273.001 | 0.98498 | 0.98366 |
| 1271.072 | 0.98464 | 0.98311 |
| 1269.144 | 0.98411 | 0.98254 |
| 1267.215 | 0.98346 | 0.98196 |
| 1265.286 | 0.98284 | 0.98136 |
| 1263.357 | 0.98236 | 0.98074 |
| 1261.429 | 0.98213 | 0.9801 |
| 1259.5 | 0.98215 | 0.97942 |
| 1257.571 | 0.98236 | 0.97876 |
| 1255.642 | 0.98261 | 0.97822 |
| 1253.713 | 0.98278 | 0.97789 |
| 1251.785 | 0.98276 | 0.97779 |
| 1249.856 | 0.98252 | 0.97791 |
| 1247.927 | 0.98211 | 0.97821 |
| 1245.998 | 0.98169 | 0.97865 |
| 1244.069 | 0.98143 | 0.97921 |
| 1242.141 | 0.98149 | 0.97984 |
| 1240.212 | 0.98193 | 0.98046 |
| 1238.283 | 0.9827 | 0.98103 |
| 1236.354 | 0.98365 | 0.98153 |
| 1234.425 | 0.98458 | 0.98199 |
| 1232.497 | 0.98528 | 0.98238 |
| 1230.568 | 0.98563 | 0.98265 |
| 1228.639 | 0.98563 | 0.98281 |
| 1226.71 | 0.98539 | 0.98288 |
| 1224.782 | 0.98508 | 0.98289 |
| 1222.853 | 0.9849 | 0.98282 |
| 1220.924 | 0.98496 | 0.98266 |
| 1218.995 | 0.9853 | 0.98241 |
| 1217.066 | 0.98583 | 0.98212 |
| 1215.138 | 0.98642 | 0.98187 |
| 1213.209 | 0.9869 | 0.9817 |
| 1211.28 | 0.98717 | 0.98156 |
| 1209.351 | 0.98721 | 0.9815 |
| 1207.422 | 0.9871 | 0.98157 |
| 1205.494 | 0.987 | 0.98184 |
| 1203.565 | 0.98705 | 0.98231 |
| 1201.636 | 0.98733 | 0.9829 |
| 1199.707 | 0.98781 | 0.98354 |
| 1197.778 | 0.98837 | 0.98421 |
| 1195.85 | 0.98886 | 0.9849 |
| 1193.921 | 0.98913 | 0.98564 |
| 1191.992 | 0.98913 | 0.98636 |
| 1190.063 | 0.98889 | 0.987 |
| 1188.135 | 0.98856 | 0.98751 |
| 1186.206 | 0.98832 | 0.98792 |
| 1184.277 | 0.98833 | 0.9882 |
| 1182.348 | 0.98865 | 0.9883 |
| 1180.419 | 0.98922 | 0.98822 |
| 1178.491 | 0.98983 | 0.98795 |
| 1176.562 | 0.99025 | 0.98757 |
| 1174.633 | 0.99029 | 0.98715 |
| 1172.704 | 0.98988 | 0.98671 |
| 1170.775 | 0.98905 | 0.98624 |
| 1168.847 | 0.98798 | 0.98575 |
| 1166.918 | 0.98697 | 0.9853 |
| 1164.989 | 0.98627 | 0.98497 |
| 1163.06 | 0.98609 | 0.98479 |
| 1161.131 | 0.98644 | 0.98472 |
| 1159.203 | 0.98718 | 0.98476 |
| 1157.274 | 0.98806 | 0.98494 |
| 1155.345 | 0.98878 | 0.98536 |
| 1153.416 | 0.98914 | 0.986 |
| 1151.488 | 0.98903 | 0.98677 |
| 1149.559 | 0.9885 | 0.98753 |
| 1147.63 | 0.98774 | 0.98821 |
| 1145.701 | 0.98703 | 0.98882 |
| 1143.772 | 0.98663 | 0.98935 |
| 1141.844 | 0.98669 | 0.98976 |
| 1139.915 | 0.98719 | 0.98996 |
| 1137.986 | 0.98794 | 0.98991 |
| 1136.057 | 0.98865 | 0.98966 |
| 1134.128 | 0.98904 | 0.98932 |
| 1132.2 | 0.98892 | 0.98891 |
| 1130.271 | 0.98825 | 0.98836 |
| 1128.342 | 0.98713 | 0.98765 |
| 1126.413 | 0.98583 | 0.98686 |
| 1124.484 | 0.98465 | 0.98613 |
| 1122.556 | 0.98386 | 0.98554 |
| 1120.627 | 0.98357 | 0.9851 |
| 1118.698 | 0.98374 | 0.98481 |
| 1116.769 | 0.98418 | 0.98469 |
| 1114.841 | 0.98466 | 0.98483 |
| 1112.912 | 0.98496 | 0.98525 |
| 1110.983 | 0.98501 | 0.98589 |
| 1109.054 | 0.98489 | 0.98668 |
| 1107.125 | 0.98479 | 0.98755 |
| 1105.197 | 0.98493 | 0.98853 |
| 1103.268 | 0.98547 | 0.98959 |
| 1101.339 | 0.98643 | 0.99065 |
| 1099.41 | 0.98764 | 0.99165 |
| 1097.481 | 0.98885 | 0.99255 |
| 1095.553 | 0.9898 | 0.99334 |
| 1093.624 | 0.99029 | 0.99405 |
| 1091.695 | 0.99032 | 0.99468 |
| 1089.766 | 0.99002 | 0.99515 |
| 1087.837 | 0.98966 | 0.99541 |
| 1085.909 | 0.9895 | 0.99553 |
| 1083.98 | 0.98977 | 0.9956 |
| 1082.051 | 0.99049 | 0.99563 |
| 1080.122 | 0.9915 | 0.9956 |
| 1078.194 | 0.9925 | 0.99549 |
| 1076.265 | 0.99322 | 0.99539 |
| 1074.336 | 0.99346 | 0.99539 |
| 1072.407 | 0.99324 | 0.99552 |
| 1070.478 | 0.99276 | 0.99576 |
| 1068.55 | 0.99232 | 0.99606 |
| 1066.621 | 0.99219 | 0.99637 |
| 1064.692 | 0.99258 | 0.99676 |
| 1062.763 | 0.99352 | 0.99724 |
| 1060.834 | 0.99481 | 0.9977 |
| 1058.906 | 0.99611 | 0.99805 |
| 1056.977 | 0.99706 | 0.99831 |
| 1055.048 | 0.9974 | 0.99856 |
| 1053.119 | 0.99706 | 0.99884 |
| 1051.19 | 0.99618 | 0.9991 |
| 1049.262 | 0.99507 | 0.99923 |
| 1047.333 | 0.9941 | 0.99918 |
| 1045.404 | 0.99357 | 0.99902 |
| 1043.475 | 0.99364 | 0.99881 |
| 1041.546 | 0.99428 | 0.99856 |
| 1039.618 | 0.99524 | 0.99816 |
| 1037.689 | 0.99616 | 0.9976 |
| 1035.76 | 0.99671 | 0.99701 |
| 1033.831 | 0.99669 | 0.9965 |
| 1031.903 | 0.99611 | 0.99612 |
| 1029.974 | 0.99516 | 0.99581 |
| 1028.045 | 0.99415 | 0.99554 |
| 1026.116 | 0.99341 | 0.99534 |
| 1024.187 | 0.99319 | 0.99527 |
| 1022.259 | 0.99354 | 0.99534 |
| 1020.33 | 0.99433 | 0.99551 |
| 1018.401 | 0.99531 | 0.99574 |
| 1016.472 | 0.99614 | 0.99604 |
| 1014.543 | 0.99658 | 0.9965 |
| 1012.615 | 0.99655 | 0.99712 |
| 1010.686 | 0.9962 | 0.99781 |
| 1008.757 | 0.99574 | 0.99844 |
| 1006.828 | 0.99543 | 0.99895 |
| 1004.899 | 0.99547 | 0.99934 |
| 1002.971 | 0.99593 | 0.99965 |
| 1001.042 | 0.99669 | 0.99983 |
| 999.1131 | 0.9975 | 0.99983 |
| 997.1843 | 0.9981 | 0.99965 |
| 995.2555 | 0.99828 | 0.99938 |
| 993.3268 | 0.99798 | 0.9991 |
| 991.398 | 0.99735 | 0.99887 |
| 989.4692 | 0.99666 | 0.99864 |
| 987.5404 | 0.99618 | 0.99837 |
| 985.6116 | 0.99611 | 0.99803 |
| 983.6828 | 0.99651 | 0.99768 |
| 981.754 | 0.99723 | 0.99739 |
| 979.8252 | 0.99799 | 0.99715 |
| 977.8964 | 0.9985 | 0.99693 |
| 975.9676 | 0.99855 | 0.99676 |
| 974.0389 | 0.99808 | 0.99677 |
| 972.1101 | 0.99722 | 0.99698 |
| 970.1813 | 0.99627 | 0.99734 |
| 968.2525 | 0.99556 | 0.99776 |
| 966.3237 | 0.99532 | 0.99818 |
| 964.3949 | 0.99565 | 0.99862 |
| 962.4661 | 0.99645 | 0.99905 |
| 960.5373 | 0.99746 | 0.99942 |
| 958.6085 | 0.9983 | 0.99961 |
| 956.6797 | 0.99863 | 0.99956 |
| 954.751 | 0.99831 | 0.99933 |
| 952.8222 | 0.99741 | 0.99903 |
| 950.8934 | 0.9962 | 0.9987 |
| 948.9646 | 0.99507 | 0.99829 |
| 947.0358 | 0.99437 | 0.99777 |
| 945.107 | 0.99428 | 0.9972 |
| 943.1782 | 0.99481 | 0.99668 |
| 941.2494 | 0.99575 | 0.99627 |
| 939.3206 | 0.99677 | 0.99591 |
| 937.3918 | 0.9975 | 0.99555 |
| 935.4631 | 0.99766 | 0.99522 |
| 933.5343 | 0.99718 | 0.99497 |
| 931.6055 | 0.99621 | 0.9948 |
| 929.6767 | 0.99506 | 0.99471 |
| 927.7479 | 0.9941 | 0.99464 |
| 925.8191 | 0.99357 | 0.99462 |
| 923.8903 | 0.99355 | 0.99468 |
| 921.9615 | 0.99391 | 0.99482 |
| 920.0327 | 0.99438 | 0.99502 |
| 918.1039 | 0.99469 | 0.99519 |
| 916.1752 | 0.9946 | 0.99532 |
| 914.2464 | 0.99404 | 0.99544 |
| 912.3176 | 0.99314 | 0.99554 |
| 910.3888 | 0.99216 | 0.99555 |
| 908.46 | 0.99137 | 0.99542 |
| 906.5312 | 0.991 | 0.99512 |
| 904.6024 | 0.99111 | 0.9947 |
| 902.6736 | 0.99157 | 0.99426 |
| 900.7448 | 0.99216 | 0.99386 |
| 898.8161 | 0.99257 | 0.99344 |
| 896.8873 | 0.99256 | 0.99297 |
| 894.9585 | 0.99207 | 0.9925 |
| 893.0297 | 0.9912 | 0.99216 |
| 891.1009 | 0.99022 | 0.99199 |
| 889.1721 | 0.98947 | 0.99186 |
| 887.2433 | 0.98916 | 0.99169 |
| 885.3145 | 0.98938 | 0.99152 |
| 883.3857 | 0.99006 | 0.99148 |
| 881.4569 | 0.99094 | 0.99162 |
| 879.5282 | 0.99174 | 0.99188 |
| 877.5994 | 0.99218 | 0.99212 |
| 875.6706 | 0.99211 | 0.99231 |
| 873.7418 | 0.99155 | 0.99254 |
| 871.813 | 0.99072 | 0.99285 |
| 869.8842 | 0.98996 | 0.99318 |
| 867.9554 | 0.98959 | 0.99342 |
| 866.0266 | 0.98975 | 0.9935 |
| 864.0978 | 0.99041 | 0.99346 |
| 862.169 | 0.99136 | 0.99345 |
| 860.2403 | 0.99229 | 0.99347 |
| 858.3115 | 0.99287 | 0.99346 |
| 856.3827 | 0.99291 | 0.99335 |
| 854.4539 | 0.99235 | 0.99316 |
| 852.5251 | 0.99138 | 0.99301 |
| 850.5963 | 0.99034 | 0.99292 |
| 848.6675 | 0.98962 | 0.99281 |
| 846.7387 | 0.98948 | 0.99268 |
| 844.8099 | 0.98991 | 0.99261 |
| 842.8811 | 0.99073 | 0.99273 |
| 840.9524 | 0.99159 | 0.99309 |
| 839.0236 | 0.99216 | 0.99363 |
| 837.0948 | 0.99217 | 0.99421 |
| 835.166 | 0.99161 | 0.99477 |
| 833.2372 | 0.99065 | 0.99531 |
| 831.3084 | 0.98965 | 0.99586 |
| 829.3796 | 0.98905 | 0.99633 |
| 827.4508 | 0.98916 | 0.99655 |
| 825.522 | 0.99001 | 0.99651 |
| 823.5932 | 0.99136 | 0.99639 |
| 821.6645 | 0.99272 | 0.9963 |
| 819.7357 | 0.99361 | 0.99629 |
| 817.8069 | 0.99367 | 0.99633 |
| 815.8781 | 0.99284 | 0.99629 |
| 813.9493 | 0.99135 | 0.99616 |
| 812.0205 | 0.98967 | 0.99602 |
| 810.0917 | 0.98832 | 0.99594 |
| 808.1629 | 0.98771 | 0.99587 |
| 806.2341 | 0.98802 | 0.9957 |
| 804.3054 | 0.98907 | 0.99543 |
| 802.3766 | 0.99042 | 0.99521 |
| 800.4478 | 0.99157 | 0.99517 |
| 798.519 | 0.99206 | 0.9953 |
| 796.5902 | 0.99171 | 0.99554 |
| 794.6614 | 0.99062 | 0.99584 |
| 792.7326 | 0.98915 | 0.99617 |
| 790.8038 | 0.9878 | 0.99664 |
| 788.875 | 0.987 | 0.99722 |
| 786.9462 | 0.98695 | 0.99775 |
| 785.0175 | 0.98756 | 0.99815 |
| 783.0887 | 0.98849 | 0.99852 |
| 781.1599 | 0.98926 | 0.99892 |
| 779.2311 | 0.98952 | 0.99928 |
| 777.3023 | 0.98907 | 0.99946 |
| 775.3735 | 0.98795 | 0.99944 |
| 773.4447 | 0.98646 | 0.9993 |
| 771.5159 | 0.98504 | 0.99909 |
| 769.5871 | 0.9841 | 0.99884 |
| 767.6583 | 0.98389 | 0.99854 |
| 765.7296 | 0.98438 | 0.99812 |
| 763.8008 | 0.98528 | 0.99764 |
| 761.872 | 0.98611 | 0.99726 |
| 759.9432 | 0.98642 | 0.99702 |
| 758.0144 | 0.98597 | 0.99687 |
| 756.0856 | 0.98481 | 0.99671 |
| 754.1568 | 0.98317 | 0.99653 |
| 752.228 | 0.98144 | 0.99643 |
| 750.2992 | 0.98012 | 0.99645 |
| 748.3704 | 0.97956 | 0.99652 |
| 746.4417 | 0.97984 | 0.99662 |
| 744.5129 | 0.98077 | 0.99673 |
| 742.5841 | 0.98189 | 0.99693 |
| 740.6553 | 0.98266 | 0.99731 |
| 738.7265 | 0.98268 | 0.99784 |
| 736.7977 | 0.98183 | 0.99837 |
| 734.8689 | 0.98031 | 0.99881 |
| 732.9401 | 0.97849 | 0.99915 |
| 731.0113 | 0.97684 | 0.99938 |
| 729.0825 | 0.97578 | 0.99945 |
| 727.1538 | 0.97552 | 0.99928 |
| 725.225 | 0.976 | 0.99892 |
| 723.2962 | 0.9769 | 0.99847 |
| 721.3674 | 0.97775 | 0.998 |
| 719.4386 | 0.97812 | 0.99756 |
| 717.5098 | 0.97774 | 0.99717 |
| 715.581 | 0.97667 | 0.99677 |
| 713.6522 | 0.97521 | 0.99635 |
| 711.7234 | 0.97377 | 0.996 |
| 709.7947 | 0.97273 | 0.99576 |
| 707.8659 | 0.97235 | 0.99557 |
| 705.9371 | 0.9726 | 0.9953 |
| 704.0083 | 0.9732 | 0.995 |
| 702.0795 | 0.9737 | 0.99483 |
| 700.1507 | 0.97371 | 0.99483 |
| 698.2219 | 0.97303 | 0.99489 |
| 696.2931 | 0.97172 | 0.99491 |
| 694.3643 | 0.97013 | 0.99489 |
| 692.4355 | 0.96873 | 0.99494 |
| 690.5068 | 0.96795 | 0.9952 |
| 688.578 | 0.96798 | 0.99566 |
| 686.6492 | 0.9688 | 0.99613 |
| 684.7204 | 0.97015 | 0.99646 |
| 682.7916 | 0.97148 | 0.99678 |
| 680.8628 | 0.97205 | 0.9976 |
| 678.934 | 0.97144 | 0.99871 |
| 677.0052 | 0.96968 | 0.99903 |
| 675.0764 | 0.96717 | 0.99854 |
| 673.1476 | 0.96456 | 0.998 |
| 671.2189 | 0.96265 | 0.99768 |
| 669.2901 | 0.962 | 0.99739 |
| 667.3613 | 0.96264 | 0.99699 |
| 665.4325 | 0.96409 | 0.99639 |
| 663.5037 | 0.96574 | 0.99561 |
| 661.5749 | 0.96698 | 0.99488 |
| 659.6461 | 0.96722 | 0.99453 |
| 657.7173 | 0.96624 | 0.99466 |
| 655.7885 | 0.96434 | 0.99452 |
| 653.8597 | 0.96216 | 0.99357 |
| 651.931 | 0.96037 | 0.99266 |
| 650.0022 | 0.95949 | 0.99245 |
| 648.0734 | 0.95979 | 0.99261 |
| 646.1446 | 0.96105 | 0.9927 |
| 644.2158 | 0.96255 | 0.99261 |
| 642.287 | 0.96347 | 0.99241 |
| 640.3582 | 0.96334 | 0.99231 |
| 638.4294 | 0.96209 | 0.99237 |
| 636.5006 | 0.96004 | 0.99245 |
| 634.5718 | 0.95782 | 0.99238 |
| 632.6431 | 0.95612 | 0.99216 |
| 630.7143 | 0.95542 | 0.99196 |
| 628.7855 | 0.95579 | 0.99209 |
| 626.8567 | 0.95693 | 0.99248 |
| 624.9279 | 0.95829 | 0.99269 |
| 622.9991 | 0.95918 | 0.99259 |
| 621.0703 | 0.95902 | 0.99231 |
| 619.1415 | 0.95762 | 0.99198 |
| 617.2127 | 0.9553 | 0.9916 |
| 615.284 | 0.9527 | 0.99098 |
| 613.3552 | 0.95058 | 0.99015 |
| 611.4264 | 0.94955 | 0.9893 |
| 609.4976 | 0.94992 | 0.98864 |
| 607.5688 | 0.95147 | 0.98825 |
| 605.64 | 0.95359 | 0.98814 |
| 603.7112 | 0.95545 | 0.988 |
| 601.7824 | 0.95628 | 0.98772 |
| 599.8536 | 0.95562 | 0.98755 |
| 597.9248 | 0.95352 | 0.98751 |
| 595.9961 | 0.95061 | 0.98738 |
| 594.0673 | 0.94783 | 0.98706 |
| 592.1385 | 0.9461 | 0.98673 |
| 590.2097 | 0.94597 | 0.98673 |
| 588.2809 | 0.9475 | 0.98715 |
| 586.3521 | 0.95014 | 0.9877 |
| 584.4233 | 0.95296 | 0.98813 |
| 582.4945 | 0.95491 | 0.98838 |
| 580.5657 | 0.95518 | 0.98853 |
| 578.6369 | 0.95353 | 0.98864 |
| 576.7082 | 0.95042 | 0.98857 |
| 574.7794 | 0.9469 | 0.98816 |
| 572.8506 | 0.94414 | 0.98755 |
| 570.9218 | 0.94297 | 0.98706 |
| 568.993 | 0.94368 | 0.9869 |
| 567.0642 | 0.94588 | 0.98705 |
| 565.1354 | 0.9486 | 0.98715 |
| 563.2066 | 0.95068 | 0.98693 |
| 561.2778 | 0.9512 | 0.98651 |
| 559.349 | 0.94977 | 0.9861 |
| 557.4203 | 0.94677 | 0.98573 |
| 555.4915 | 0.94325 | 0.98522 |
| 553.5627 | 0.94049 | 0.98447 |
| 551.6339 | 0.93944 | 0.98365 |
| 549.7051 | 0.94038 | 0.98313 |
| 547.7763 | 0.9428 | 0.98298 |
| 545.8475 | 0.94568 | 0.98294 |
| 543.9187 | 0.94779 | 0.98272 |
| 541.9899 | 0.94812 | 0.98232 |
| 540.0611 | 0.94637 | 0.98205 |
| 538.1324 | 0.94298 | 0.98211 |
| 536.2036 | 0.93911 | 0.98216 |
| 534.2748 | 0.93621 | 0.98187 |
| 532.346 | 0.93544 | 0.98135 |
| 530.4172 | 0.93718 | 0.98092 |
| 528.4884 | 0.94087 | 0.98081 |
| 526.5596 | 0.94531 | 0.98087 |
| 524.6308 | 0.949 | 0.98076 |
| 522.702 | 0.95063 | 0.98047 |
| 520.7733 | 0.94952 | 0.98033 |
| 518.8445 | 0.946 | 0.98064 |
| 516.9157 | 0.94126 | 0.98126 |
| 514.9869 | 0.9369 | 0.98176 |
| 513.0581 | 0.93443 | 0.98178 |
| 511.1293 | 0.93473 | 0.98154 |
| 509.2005 | 0.93763 | 0.98156 |
| 507.2717 | 0.94206 | 0.98196 |
| 505.3429 | 0.94651 | 0.98241 |
| 503.4141 | 0.94954 | 0.98258 |
| 501.4854 | 0.95016 | 0.98263 |
| 499.5566 | 0.94815 | 0.98303 |
| 497.6278 | 0.94429 | 0.98383 |
| 495.699 | 0.94015 | 0.98463 |
| 493.7702 | 0.93748 | 0.98499 |
| 491.8414 | 0.93748 | 0.98498 |
| 489.9126 | 0.94042 | 0.98507 |
| 487.9838 | 0.94535 | 0.98559 |
| 486.055 | 0.95053 | 0.98647 |
| 484.1262 | 0.95418 | 0.98722 |
| 482.1975 | 0.95507 | 0.98748 |
| 480.2687 | 0.95286 | 0.98757 |
| 478.3399 | 0.94834 | 0.98799 |
| 476.4111 | 0.94321 | 0.98846 |
| 474.4823 | 0.93954 | 0.98831 |
| 472.5535 | 0.93893 | 0.98741 |
| 470.6247 | 0.94185 | 0.98634 |
| 468.6959 | 0.94756 | 0.98584 |
| 466.7671 | 0.95419 | 0.98612 |
| 464.8383 | 0.9594 | 0.98676 |
| 462.9096 | 0.96132 | 0.98713 |
| 460.9808 | 0.95916 | 0.98712 |
| 459.052 | 0.95348 | 0.98714 |
| 457.1232 | 0.94606 | 0.98758 |
| 455.1944 | 0.93935 | 0.98812 |
| 453.2656 | 0.93557 | 0.98797 |
| 451.3368 | 0.93581 | 0.98704 |
| 449.408 | 0.93955 | 0.98626 |
| 447.4792 | 0.94497 | 0.98638 |
| 445.5504 | 0.94976 | 0.98712 |
| 443.6217 | 0.95193 | 0.98765 |
| 441.6929 | 0.95048 | 0.98756 |
| 439.7641 | 0.94577 | 0.98718 |
| 437.8353 | 0.93933 | 0.98702 |
| 435.9065 | 0.93363 | 0.98678 |
| 433.9777 | 0.93119 | 0.98587 |
| 432.0489 | 0.93337 | 0.98454 |
| 430.1201 | 0.93975 | 0.98318 |
| 428.1913 | 0.94829 | 0.98211 |
| 426.2626 | 0.95605 | 0.98175 |
| 424.3338 | 0.96027 | 0.98181 |
| 422.405 | 0.95938 | 0.98198 |
| 420.4762 | 0.95383 | 0.98268 |
| 418.5474 | 0.94546 | 0.9843 |
| 416.6186 | 0.93782 | 0.98691 |
| 414.6898 | 0.93226 | 0.98986 |
| 412.761 | 0.92929 | 0.99246 |
| 410.8322 | 0.92869 | 0.9946 |
| 408.9034 | 0.92961 | 0.9946 |
| 406.9747 | 0.93082 | 0.9946 |
| 405.0459 | 0.93082 | 0.9946 |
| 403.1171 | 0.93082 | 0.9946 |
| 401.1883 | 0.93082 | 0.9946 |
| 399.2595 | 0.93082 | 0.9946 |
